# Double-truncated version of OsGADs leads to higher GABA accumulation and stronger stress tolerance in *Oryza sativa* L. var. *japonica*

**DOI:** 10.1007/s00299-025-03477-y

**Published:** 2025-04-08

**Authors:** Ummey Kulsum, Nadia Akter, Kazuhito Akama

**Affiliations:** 1 Graduate School of Natural Science and Technology, Shimane University, 1060, Nishikawatsu, Matsue, Shimane 690-8504 Japan; 2 Present Address: Genetic Resources and Seed Division, Bangladesh Rice Research Institute, Gazipur, 1701 Bangladesh

**Keywords:** Glutamate decarboxylase, GABA, Genome-editing, Abiotic stress, *Oryza sativa* L.

## Abstract

**Key message:**

Calmodulin binding domain truncation from OsGAD1 and OsGAD3 resulted in enhanced GABA accumulation, upregulated stress related genes, and improved tolerance to multiple abiotic stresses.

**Abstract:**

Rice (*Oryza sativa* L.), a critical crop for global food security, faces significant challenges from abiotic stresses. Gamma-aminobutyric acid (GABA), synthesized by glutamate decarboxylase (GAD), plays a vital role in stress tolerance. Truncating the calmodulin-binding domain (CaMBD) in GAD enzymes enhances GAD activity and GABA production. In this study, we developed a hybrid line, Hybrid #78, by crossing two genome-edited lines, OsGAD1ΔC #5 and OsGAD3ΔC #8, with truncated CaMBD in OsGAD1 and OsGAD3, respectively. Hybrid #78 demonstrated significantly improved survival rates in cold (25%), salinity (33%), flooding (83%), and drought (83%) stress conditions, compared with wild-type Nipponbare (0–33%), OsGAD1∆C #5 (0–66%), and OsGAD3∆C #8 (0–50%). Hybrid #78 showed the highest GABA levels during stress, with increases of 3.5-fold (cold), 3.9-fold (salinity), 5-fold (flooding), and 5-fold (drought) relative to wild-type Nipponbare and up to 2-fold higher than that of the parent lines. RNA-seq analysis from shoot tissues in control conditions identified 975 differentially expressed genes between Hybrid #78 and wild-type Nipponbare, with 450 genes uniquely expressed in the hybrid. Kyoto Encyclopedia of Genes and Genomes (KEGG) enrichment revealed that upregulation in nitrogen metabolism pathways likely contributes to enhanced GABA synthesis via increased glutamate production. Hybrid #78 also showed broader gene expression variability, suggesting enhanced adaptability to stress, especially upregulation of stress-related genes, such as *OsDREB*, *OsHSP70*, and *OsNAC3*. These findings highlight the potential of CaMBD truncation in OsGAD1 and OsGAD3 to develop rice lines with increased GABA accumulation and resilience to multiple abiotic stresses.

**Supplementary Information:**

The online version contains supplementary material available at 10.1007/s00299-025-03477-y.

## Introduction

Rice (*Oryza sativa* L.) is a critical global food crop that plays a significant role in providing sustenance to a substantial portion of the world's population. During the 2023/24 crop year, global rice consumption reached approximately 523.8 million metric tons (Total Global Rice Consumption 2024/25, n.d.), supplying around 21% of the world's per capita energy intake and 15% of per capita protein consumption (Rice Production Course, n.d.). In addition, it offers tremendous potential as a model organism for investigating how crops respond to abiotic stressors (Radha et al. 2023). Understanding the mechanisms underlying the resilience of rice to environmental pressures is of utmost importance, given its direct impact on both food security and the sustainability of agricultural practices. Abiotic stresses, such as cold temperature, flooding, high salinity levels, and drought, pose substantial challenges to rice cultivation worldwide. These stress factors exert distinct physiological effects on rice plants, often resulting in significant reductions in crop yield. For example, high salinity levels can disrupt osmotic regulation and ion balance, affecting water uptake and nutrient absorption (Kruthika and Jithesh 2023; Nagarajan et al. 2022), leading to annual yield losses of 30–50% (Zheng et al. 2021). Drought severely affects water availability, leading to reduced photosynthesis and impaired plant growth (Sandeep and Godi 2023), affecting yield losses of 20–50% during critical growth stages (Amin et al. 2022). Understanding the responses of rice to these abiotic stressors is essential for developing effective strategies to mitigate their negative impacts. Studying the molecular, physiological, and biochemical factors that contribute to the ability of rice to withstand abiotic stress will aid in the identification of crucial genes and pathways associated with stress tolerance.

Glutamate decarboxylase (*GAD*) genes encode enzymes that play a crucial role in stress tolerance in plants (Islam et al. 2024; Zheng et al. 2024). These enzymes catalyze the conversion of glutamate into γ-aminobutyric acid (GABA), a metabolite that accumulates significantly in environmental stress conditions, such as salt, drought, cold, and flooding stress (Srivastava et al. 2021). In addition, the calmodulin (CaM)-binding domain (CaMBD) in the C-terminal region of plant GADs plays a crucial role in regulating the activity of GAD enzymes. In normal conditions, the CaMBD acts as an inhibitory regulator of GAD activity (Baum et al. 1993). Through binding of calcium (Ca^2+^)/CaM to CaMBD in GADs, the activity of GAD enzymes is restored, thereby enhancing the production of GABA. Consequently, when the CaMBD is truncated, this inhibitory effect is lifted, leading to an increase in GAD activity, resulting in a constitutively active enzyme that no longer requires Ca^2^⁺/CaM binding to be activated. Thus, the conversion of glutamate to GABA is enhanced, leading to higher levels of GABA accumulation in plant cells (Baum et al. 1996; Akama et al. 2020; Akter et al. 2024). The accumulation of GABA serves multiple functions that contribute to stress tolerance. First, GABA helps in maintaining cellular pH and osmotic balance, crucial factors for proper cell functioning in stress conditions (Kumari et al. 2024; Hasan et al. 2021). In addition, GABA acts as a signaling molecule that modulates ion channels, such as ALMT (aluminum-activated malate transporter) and GORK (guard cell outward-rectifying K⁺ channel), which is important for osmotic adjustment and ionic homeostasis in plants (Ghosh 2020). Furthermore, GABA is known to have a protective effect against oxidative stress, which is a common consequence of adverse environmental conditions (Suhel et al. 2023). In addition, GABA priming has been shown to offer protection against abiotic stresses in many plants by enhancing leaf turgor, boosting osmolyte levels, and minimizing oxidative damage through the activation of antioxidant mechanisms (Hayat et al. 2023).

Previous studies aimed at enhancing stress tolerance in rice have primarily focused on genes, such as *OsDREB*, *OsSALP1*, and *OsSAPK2*, utilizing approaches, such as overexpression and gene silencing (Liu et al. 2023; Shim et al. 2018; Lou et al. 2017; Yuan et al. 2016; Zhang et al. 2013; Chen et al. 2008). In contrast, there has been relatively little focus on OsGADs, despite their central role in GABA metabolism, which is crucial for rapid stress responses (Ansari et al. 2021; Lee et al. 2021). In recent years, the genes *OsGAD1* and *OsGAD3* derived from rice have attracted considerable interest due to their involvement in the potential for enhancing crop productivity. Specifically, *OsGAD1,* which is located on chromosome 8 (AB056060) and encodes a protein of 501 amino acids, has been extensively studied for its impact on awn development and yield in Kam Sweet Rice (Luo et al. 2024). *OsGAD3*, another member of this family, predominantly expressed in seeds, has been targeted for its CaMBD to increase GABA content in grains. By deleting the CaMBD from OsGAD3 using CRISPR/Cas9, Akama et al. (2020) successfully enhanced GABA accumulation and increased seed weight and protein content. *OsGAD3* (AK071556) is found on rice chromosome 3 and encodes a protein of 492 amino acids. Although *OsGAD1* and *OsGAD3* have been explored for their roles in rice development and nutritional enhancement, their direct involvement in abiotic stress tolerance through genome editing has not been reported. However, our previous study (Akter et al. 2024) already reported that truncating the CaMBD of OsGAD4 resulted in a significant increase in GABA accumulation and also enhanced tolerance to abiotic stress. Moreover, putative OsGAD1 and OsGAD3 possess the typical CaMBD, and at least the removal of CaMBD of OsGAD3 enhanced GAD activity and led to increased GABA accumulation (Akama et al. 2020) suggesting a potential role in abiotic stress tolerance, similar to OsGAD4.

Recent advances in CRISPR/Cas9 technology for editing genomes have paved the way for the precise manipulation of specific genes. The objective of this study was to utilize CRISPR/Cas9 to selectively edit the genes *OsGAD1* and *OsGAD3* to directly enhance GABA production, aiming to take advantage of this naturally occurring stress response mechanism more effectively. By enhancing the natural stress response mechanism of GABA accumulation, this study aimed to develop new varieties of rice that possess increased resilience to challenging environmental conditions. Traditionally, the focus of research in this field has mainly been on the editing of individual genes responsible for stress tolerance. However, this study not only concentrated on editing the genes *OsGAD1* and *OsGAD3* individually but also on producing a hybrid line by crossing the two genome-edited lines. Taking advantage of hybridization provides the opportunity to combine the advantageous traits from both parent lines, resulting in progeny that exhibit improved tolerance to environmental stressors. Therefore, the purpose of this study was to preciously compare GABA content and stress tolerance among wild type (WT), parental, and hybrid lines. By taking advantage of the potential of gene-editing technologies and hybridization approaches, this research will contribute to the development of novel rice varieties that are better equipped to withstand harsh environmental conditions and partially elucidate the molecular mechanism underlying abiotic stress tolerance.

## Materials and methods

### Plant materials and growth conditions

*Oryza sativa* L. var. *japonica* cv. Nipponbare (Ni) was used in this study as a control plant. For in vitro tissue culture, rice seeds were dehulled using automatic rice husker TR-260 (Kett Electric Laboratory Co. Ltd., Tokyo, Japan), followed by immersion in 70% (v/v) ethanol for 1 min then rinsed with double distilled water (ddH_2_O). Afterward, seeds were surface sterilized using 50% (v/v) bleach solution (Kao Co. Ltd, Tokyo, Japan) for 30 min with gentle shaking, then washed five times with ddH_2_O. The seeds were then transferred to 0.5 × Murashige and Skoog (MS) agar medium (Murashige & Skoog 1962) to germinate in a growth chamber at 25 °C (SANYO, Osaka, Japan) with illumination by white fluorescent tubes for 2 weeks. Later, the seedlings were moved into soil (JA, EPOCH Co., Ltd, Izumo, Japan) in a growth room with 16 h/8 h light/dark conditions at the same temperature.

### Production of CaMBD-truncated OsGAD1 genome-edited lines

Single guide RNAs (gRNAs) were generated using the CRISPR-P program (http://crispr.hzau.edu.cn/CRISPR2/) to truncate the C-terminal region of *OsGAD1*. Three gRNAs were derived from the 3’-terminal coding region of *OsGAD1* shown in Fig. S1: gRNA-F1 and gRNA-F2, located upstream of the CaMBD domain, and gRNA-R1, located downstream. Each gRNA target sequence (Table S4), composed of 20 nucleotides, was synthesized and annealed to create double-stranded DNA. These were then inserted into the *Bbs*I site of the gRNA cloning vector pU6gRNA, designated as pU6gRNA_F1, pU6gRNA_F2, and pU6gRNA_R1, following the method described by Mikami et al. (2015). The gRNA expression cassette carrying the gRNA-R1 fragment, generated by digesting plasmid pU6gRNA_R1 with *Pvu*II and *Asc*I*,* was subsequently incorporated into pU6gRNA_F1 or pU6gRNA_F2 using the *Eco*RV and *Asc*I sites. This resulted in the formation of two constructs: pU6gRNA_F1 _R1 and pU6gRNA_F2 _R1, respectively. These constructs were then independently introduced into the Ti plasmid pZH_gYSA_MMCas9 via its *Asc*I and *Plm*I sites to enable rice transformation. Next, the binary vector was introduced into *Agrobacterium tumefaciens* strain EHA105 (Hood et al. 1993) by electroporation. The rice calli were transformed using *Agrobacterium*-mediated transformation and then selected on N6D medium supplemented with 50 mg/L hygromycin B in accordance with the rice transformation protocol (Ozawa 2009). The selected calli were regenerated into plants, and four transgenic lines were finally selected as candidates as successful genome-edited lines.

### Analysis of OsGAD1ΔC rice line

Six rice grains from each of the four transgenic lines (T_1_ generation) were pooled and finely ground using a MicroSmash (Tomy, Tokyo, Japan). A 20 mg sample of the resulting powder was used for the isolation of free amino acids, following the method described by Akama et al. (2009) with 8% (v/v) trichloroacetic acid (TCA). Simultaneously, a portion of the rice powder was utilized for the extraction of total DNA using the cetyltrimethylammonium bromide (CTAB) method (Murray and Thompson 1980). The target sequence coding region of *OsGAD1* in the genome-edited rice plants was PCR-amplified using specific primer sets (Table S5) to screen for successful genome editing in the transgenic lines.

### Genome-edited hybrid line establishment

In this study, we utilized two genome-edited lines: OsGAD1ΔC #5*,* which was generated through CRISPR/Cas9-mediated genome editing, and OsGAD3ΔC #8*,* which had been developed previously (Akama et al. 2020). These lines were characterized by the C-terminal truncation of OsGAD1 and OsGAD3, respectively. A single cross-hybridization strategy was employed to combine the genetic alterations of both lines into a singular hybrid line. Specifically, the homozygous OsGAD1ΔC #5 line, was designated as the female parent, and the homozygous OsGAD3ΔC #8 line served as the male parent. This crossbreeding led to the creation of a heterozygous hybrid line with the combined C-terminal truncations of OsGAD1 and OsGAD3. The hybrid line was self-pollinated to select homozygous OsGAD1ΔC #5 and OsGAD3ΔC #8 line, yielding Hybrid #78 line by PCR screening described below.

### PCR screening of genome-edited hybrid line

For the screening of hybrid lines, genomic DNA was extracted from the leaves (3 × 3 cm) using DNA isolation buffer (200 mM Tris–HCl, pH 7.5, 250 mM NaCl, 25 mM EDTA, 0.5% SDS). Isolated DNA was used for PCR amplification using EmeraldAmp® PCR Master Mix (TAKARA, Japan) followed by 30 cycles of denaturation at 98 °C for 10 s, annealing at 55 °C for 30 s, and extension at 72 °C for 30 s. Target-specific primers for *OsGAD1* and *OsGAD3* were employed to amplify the regions of interest (Table S5).

### Abiotic stress treatments: determination of biomass loss and survival rate

To investigate the effects of abiotic stress treatments, seedlings were initially cultivated for 2 weeks in optimal growth conditions in 0.5 × MS agar media. After this initial growth period, seedlings were exposed to various abiotic stress conditions for specified durations to assess their responses.

For cold stress, 15-day-old seedlings were subjected to 4 °C in 0.5 × MS media to simulate a cold-stress environment. Flooding stress was induced by submerging 15-day-old seedlings in 1 × liquid MS media to mimic waterlogged conditions. Drought stress was simulated by removing 16-day-old seedlings from MS media ensuring no residual media remained on the roots and placing them on plastic plates to replicate limited water availability. Salinity stress was induced by exposing 14-day-old seedlings to a 150 mM NaCl solution. Samples were collected at various timepoints during these treatments to analyze molecular and biochemical changes.

For biomass loss assessment, seedlings were exposed to cold, flooding, and salinity stress conditions for 2 days, whereas drought stress was applied for 6 h. Afterward, fresh weight data was recorded, and the seedlings were dried at 65 °C for 24 h to measure dry weight.

A separate set of experiments was conducted with similar stress conditions to assess survival rates but for different periods. For cold stress, seedlings were exposed to 4 °C for 5 days. Flooding stress involved submerging seedlings in MS liquid media for 3 days. For drought stress, seedlings were left on plastic plates until they lost about 65% of their fresh weight. For salinity stress, seedlings were exposed to 150 mM NaCl for 2 days. After each stress treatment, seedlings were rehydrated in normal water for 3 h and then transferred to soil for 18 days for recovery in normal conditions. The survival rates were evaluated based on the number of plants that survived this recovery period.

### Amino acid assessment by GABase assay and gas chromatography–mass spectrometry

For the isolation of amino acids in various stress-treated seedlings, shoot and root tissues were collected from control seedlings (without stress treatments) as well as those subjected to cold, flooding, drought, and salinity stresses. In specific time intervals (1, 3, 6, 12, and 24 h), approximately 30 mg of sample was obtained. The collected samples were immediately frozen in liquid nitrogen and subsequently homogenized. Following this, 8% (v/v) TCA was added to the samples, in accordance with a previously described protocol (Akama et al. 2009). The supernatant was obtained by mixing and centrifugation, which was then combined with diethyl ether. The mixture underwent further mixing and centrifugation steps. After repeating this process, the supernatant containing the diethyl ether was discarded, and the remaining solution was air-dried to obtain the amino acid solution (Akama and Takaiwa 2007).

The GABase assay was then conducted to measure the concentration of GABA. The assay involved a reaction mixture consisting of 0.1 M pyrophosphate buffer (pH 8.4), 60 mM 2-mercaptoethanol, 60 mM α-ketoglutarate, 50 mM NADP^+^, and 0.005 units of GABase enzyme obtained from Sigma. In addition, the mixture was added to an isolated amino acid sample and GABA standards as references. The reaction mixture was incubated at 37 °C for 1 h. Subsequently, the quantification of GABA content was performed using a fluorescence plate reader (Genios FL, TECAN, Salzburg, Austria). The fluorescence readings of the samples were compared against a GABA standard curve to determine the concentration of GABA present (Akama et al. 2009).

Free amino acid contents were determined using gas chromatography/mass spectrometry (GC/MS), in accordance with a previously established protocol as described by Kowaka et al. 2015. For sample preparation, the dedicated EZ:Faast™ kit provided by Phenomenex (Torrance, CA, USA) was used to derivatize the samples. Subsequently, the derivatized samples were subjected to analysis using a GC/MS-QP2010 system (Shimadzu Co. Ltd., Kyoto, Japan), which featured electronic pressure control and was equipped with a split capillary inlet. In the GC/MS analysis, a 1-μl aliquot of each sample was injected into the system in split mode. The injection temperature was set at 280 °C, and a ZB-AAA column (length 10 m, diameter 0.25 mm; Phenomenex) was used for the chromatographic separation. The gas flow rate of helium was maintained at 3.0 ml/min throughout the analysis.

### RNA isolation and RT-qPCR

Total RNA was isolated from shoot and root tissues of the seedlings using an ISOSPIN Plant RNA kit (Nippon Gene Co., Ltd., Tokyo, Japan) in accordance with the manufacturer’s instructions. cDNAs were synthesized from 1 μg of template RNA using reverse transcriptase (ReverTra Ace, TOYOBO, Osaka, Japan). To analyze gene expression, reverse transcription quantitative real-time polymerase chain reaction (RT-qPCR) was conducted using an ECO real-time PCR system (PCR max, United Kingdom). The expression levels of target genes were quantified using the 2^−∆∆CT^ method (Livak and Schmittgen 2001), with TATA-binding protein 2 (TBP-2) serving as an internal control (Zhu et al. 2012) for normalization. The primer sequences are shown in Table S4.

### RNA sequencing

RNA sequencing was performed using total RNA isolated from shoot tissue via Illumina platforms, based on the sequencing mechanism by synthesis. This service was provided by Nippon Genetics Co., Ltd. (https://n-genetics.com/ngs/) as a contract service. Poly-T oligo-attached magnetic beads were utilized to purify messenger RNA from total RNA. Following fragmentation, first strand cDNA was created using random hexamer primers. Subsequently, second strand cDNA synthesis was carried out using dUTP for the directional library, as described by Parkhomchuk et al. (2009).

The initial step involved processing the raw data in fastq format using custom perl scripts. Reference genome (ensemblplants_oryza_sativa_japonica_group_irgsp_1_0_gca_001433935_1, dosa: 4530) and gene model annotation files were obtained directly from the genome website. To align the paired-end clean reads to the reference genome, an index was built using Hisat2 v2.0.5 (Mortazavi et al. 2008), and alignment was performed using the same program. The reads mapped to each gene were counted using FeatureCounts v1.5.0-p3 (Liao et al. 2014). The FPKM value for each gene was then calculated by taking into account of the gene length and the number of reads mapped to it. Differential expression analysis between two conditions or groups was carried out using the DESeq2R package (v1.20.0) (Anders and Huber 2010). To identify any enriched Gene Ontology (GO) terms in the differentially expressed genes, the cluster profile R package was used. This analysis corrected any bias introduced due to the gene length. GO terms with a corrected *P* value less than 0.05 were considered as significantly enriched by the differentially expressed genes.

### Statistical analysis

All data are presented as the mean ± standard deviation (SD) from three biological replicates. Statistical analysis was performed using Student’s *t* test for pairwise comparisons and one-way ANOVA for multiple group comparisons in Microsoft Excel. Statistical significance was defined as ^*^P < 0.05 and ^**^P < 0.01.

## Results and discussion

### Production of CaMBD-truncated OsGAD1 rice line by genome editing

The rice genome contains a total of five genes encoding OsGAD isoforms (International Rice Genome Sequencing Project 2005), which are involved in the process of decarboxylation of glutamate to produce GABA. The C-terminal region of these isoforms has a CaMBD that is referred to as the autoinhibitory domain. This domain plays a significant role in the regulation of GAD activity in response to intracellular calcium levels via the Ca^2+^/CaM complex (Trobacher et al. 2013; Akama et al. 2001). Among the OsGAD family, OsGAD2 is unique in that it lacks the conventional CaMBD, rendering it unable to bind Ca^2+^/CaM, and thus, it is not modulated by Ca^2+^/CaM in the same manner as its counterparts (Akama and Takaiwa 2007). Conserved residues (Fig. S2a), such as tryptophan (W) and lysine (K), in OsGAD1, OsGAD3, and OsGAD4 form functional CaMBDs, characterized by amphipathic helixes (Fig. S2b). In contrast, OsGAD2 lacks this W residue and the K cluster (Fig. S2a, b), which play essential roles in hydrophobic and electrostatic interactions, respectively. These interactions are vital for the effective binding of CaM (Arazi et al. 1995), suggesting that OsGAD2 does not possess a functional CaMBD. Conversely, OsGAD1, OsGAD3, and OsGAD4 have been shown to contain the authentic CaMBD from an in vitro binding assay (Akama et al. 2001, 2020; Akter et al. 2024), and truncation of the C-terminal region of GAD3 and GAD4 showed higher enzymatic activities (Akama et al. 2020; Akter et al. 2024), so it is expected that GAD1 may exhibit a similar characteristic. This implies that the CaMBD of OsGAD1 likely also functions as an autoinhibitory domain.

To remove the coding region for the C-terminal CaMBD of OsGAD1, gRNAs were strategically designed to target regions upstream and downstream of the CaMBD-encoding region, as shown in Fig. S1a. Rice transformation of calli accompanied with genome editing resulted in the production of four distinct OsGAD1ΔC mutant lines, each exhibiting some alterations in their nucleotide sequences (Fig. S1b) and deduced amino acid sequences (Fig. S1c). After the establishment of these homozygote lines, the GABA content and brown rice weight were measured in the T_1_ generation mutant lines (Table S1). Among these lines, OsGAD1ΔC #5 exhibited nearly complete removal of the CaMBD from OsGAD1 and displayed the highest GABA content compared with wild-type (WT) Ni and other genome-edited lines. As a result, OsGAD1ΔC #5 was selected for further analysis.

### CaMBD-truncated OsGAD1, OsGAD3 and hybrid line

**Fig. 1:**
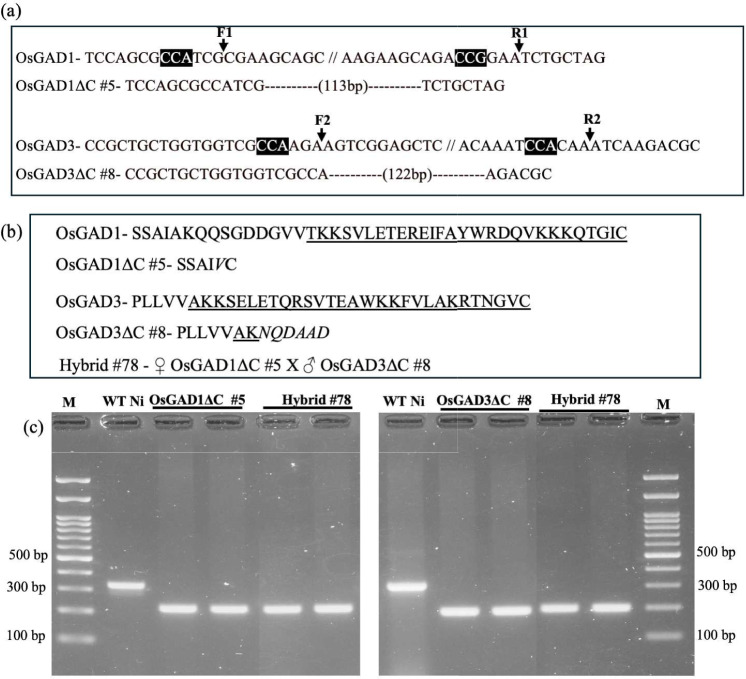
Establishment of CaMBD-truncated OsGAD1 and OsGAD3 lines by CRISPR/Cas9 genome editing along with their hybrid line. (**a)** OsGAD1ΔC #5 contains a 113 bp deletion within the target region of wild-type (WT) *OsGAD1*. The positions of F1 and R1 indicate the upstream and downstream CRISPR/Cas9 putative cleavage sites, respectively, within the targeted CaMBD region of OsGAD1; OsGAD3ΔC #8 (Akama et al. 2020) containing a 122 bp deletion; F2 and R2 shows the upstream and downstream CRISPR/Cas9 putative cleavage sites in the OsGAD3 target site, accordingly; letters highlighted in the black box within the WT sequence represents the PAM complementary sequence; (**b)** amino acid sequences in WT OsGAD1, OsGAD3, and CaMBD-truncated OsGAD1ΔC #5 and OsGAD3ΔC #8. Underlined letters indicate the CaMBD sequence in the WT C-terminal region. Italics letters indicate the additional amino acids produced by genome editing. Hybrid line #78 indicates the combination of a cross between the OsGAD1 and OsGAD3 genome-edited lines. (**c)** PCR amplification of OsGAD1 and OsGAD3 in the hybrid line had shorter products, with a 198 bp fragment for OsGAD1 and a 208 bp fragment for OsGAD3, indicating 113 bp and 122 bp deletions in the CaMBD regions of OsGAD1 and OsGAD3, respectively. These truncated products were consistent with those observed in the parental lines OsGAD1ΔC #5 and OsGAD3ΔC #8, compared with the WT Nipponbare (WT Ni). 100 bp DNA marker (indicated with the letter M) was used to identify the PCR product sizes

Figure 1a illustrates the genomic modifications in OsGAD1ΔC and OsGAD3ΔC, where targeted deletions of 113 bp and 122 bp, respectively, were introduced at the C-terminal regions through CRISPR/Cas9 genome editing. These deletions resulted in truncated proteins with shorter amino acid sequences, effectively removing the CaMBD, as shown in Fig. 1b, thereby altering the regulatory mechanism of these enzymes and potentially affecting their functionality in GABA production.

To explore the combined effects of these truncations, a hybrid line, Hybrid #78, was generated through crossbreeding of the homozygous lines OsGAD1ΔC #5 (female parent) and OsGAD3ΔC #8 (male parent) (see Materials and Methods). This cross was designed to merge the truncated versions of both OsGAD1 and OsGAD3 into each single genetic line. PCR analysis confirmed the successful incorporation of both truncated genes within the hybrid line, as shown in Fig. 1c. It was expected that double-truncated versions of OsGAD1 and OsGAD3 isoforms may express at least additive effects on GABA metabolism and stress responses.

Agronomic traits measured in OsGAD1ΔC #5, OsGAD3ΔC #8, and Hybrid #78 (Table S2) revealed a slight increase in leaf blade size and culm length (cm) compared with WT Ni. However, no significant phenotypic changes were observed between the genome-edited lines and WT. Furthermore, RT-qPCR analysis of the four *OsGAD* genes (Fig. S3) showed a slight upregulation of *OsGAD1* expression in the leaf (Fig. S3a) and *OsGAD3* expression in the root (Fig. S3c) in the hybrid line, with expression levels like those in the parent lines compared with WT Ni. In addition, the expression levels of the three *GABA-T* genes (Fig. S4) remained consistent with those in WT Ni across OsGAD1ΔC #5, OsGAD3ΔC #8, and Hybrid #78, indicating that the genome editing did not significantly alter the expression of these genes, maintaining a similar expression profile to WT. As expected, Table S3 clearly shows GABA accumulated more in Hybrid #78 than parental lines OsGAD1ΔC #5 and OsGAD3ΔC #8, indicating an additive effect of hybrid line on GABA content in vegetative tissues.

### GABA accumulation in response to abiotic stresses

*OsGAD1* and *OsGAD3* have been reported to be upregulated in response to cold, salinity, flooding, and drought stress conditions, as documented in the Transcriptome Encyclopedia of Rice (TENOR) database (https://tenor.dna.affrc.go.jp/) (Kawahara et al. 2016; Fig. S5). This upregulation was consistent with the hypothesis that higher expression levels of these genes should correlate with increased GABA accumulation, a phenomenon that has been observed in various plant species in stress conditions (Zhou et al. 2024; Sita and Kumar 2020; Kreps et al. 2002). Notably, this accumulation was enhanced in the CaMBD-truncated version of OsGAD4 (Akter et al. 2024). Based on these findings, we hypothesized that the upregulation of *OsGAD1* and *OsGAD3* may enhance GAD activity, leading to increased GABA accumulation in abiotic stress conditions. To test this hypothesis, we subjected OsGAD1ΔC #5, OsGAD3ΔC #8, and Hybrid #78 to four different abiotic stress conditions: cold, salinity, flooding, and drought and quantified the GABA concentration in these genome-edited lines over various time periods, comparing the results to WT Ni.

**Fig. 2:**
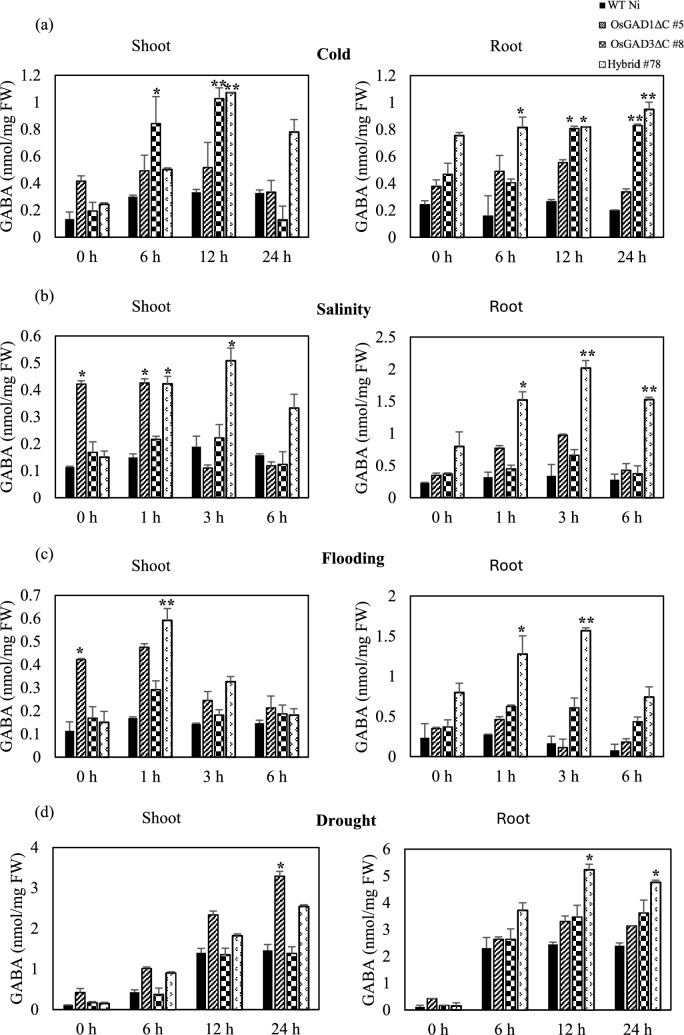
Quantitative analysis of GABA content in response to abiotic stresses in rice seedlings. (**a**) **Cold stress response**: GABA levels were assessed in 16-day-old seedlings of WT-Ni, OsGAD1ΔC #5, OsGAD3ΔC #8, and Hybrid #78 after exposure at 4 °C. Samples were collected at intervals of 6, 12, and 24 h after stress induction. (**b**) **Salinity stress response**: seedlings aged 14 days were subjected to 150 mM NaCl solution, with tissue samples harvested at 1, 3, and 6 h. ( **c**) **Flooding stress response**: 15-day-old seedlings were fully submerged in liquid Murashige and Skoog (MS) media, mirroring the time intervals used for salinity stress, to monitor GABA synthesis in hypoxic conditions. (**d**) **Drought stress response**: seedlings aged 16 days were removed from MS media and placed on plastic plates to simulate drought conditions, with sample collection at 6, 12, and 24 h after stress application. The control (0 h) represents baseline GABA content in non-stress conditions. The error bars denote the mean ± standard deviation (SD) based on three biological replicates (n = 3). FW = fresh weight. Statistical significance was determined by comparing the values of each rice line with the wild type in identical stress conditions. Asterisks denote significant differences (*P < 0.05, **P < 0.01)

When exposed to cold stress at 4 °C (Fig. 2a), the GABA content in the shoot tissues of all rice lines, particularly OsGAD1ΔC #5, OsGAD3ΔC #8, and Hybrid #78, increased up to the 12 h point before subsequently declining. Hybrid #78 showed the greatest increase, with GABA levels approximately 3.5 times higher than those in WT Ni. In root tissues, a significant rise in GABA levels was noted across the genome-edited lines, peaking at 24 h for OsGAD3ΔC #8 and Hybrid #78, whereas a decrease was observed in OsGAD1ΔC #5. In salinity stress conditions (Fig. 2b) using 150 mM NaCl solution for 1, 3, and 6 h, GABA levels changed in the shoot and root tissues of 14-day-old seedlings. The OsGAD1ΔC #5 and OsGAD3ΔC #8 lines did not show substantial increases in GABA levels, whereas Hybrid #78 displayed a marked increase, especially in root tissue at the 3 h mark, reaching approximately 3.9 times the levels in WT Ni and 2 times those in the parent line’s root tissue. Similar GABA accumulation has been recorded in other plant species in salinity stress conditions, including a 1.5-fold increase in *Arabidopsis* (Renault et al. 2010), a 1.5-fold increase in tomato (Wu et al. 2020), an 11–17 fold increase in soybean (Xing et al. 2007), and a 3–7 fold increase in wheat (Al-Quraan and Al-Omari 2017). During flooding stress (Fig. 2c), the GABA content increased initially in shoot tissues of all genome-edited lines before declining after 1 h. In root tissues, OsGAD1ΔC #5 exhibited a slight initial rise before a significant decrease, whereas Hybrid #78 showed the highest GABA concentration at the 3 h mark, at about 5 times higher than WT Ni and 2 times higher than the parental lines. For drought stress simulation (Fig. 2d), where 16-day-old seedlings were removed from MS media and placed on plastic plates for 6, 12, and 24 h, GABA levels progressively increased in both shoot and root tissues of all rice lines, with the most significant accumulation in Hybrid #78 in root tissue at 12 h. Hybrid #78 consistently maintained higher GABA levels compared with WT Ni and the parental lines, indicating a stronger stress response capacity.

Our results indicated a significant enhancement in GABA accumulation in the genome-edited lines, with the hybrid line exhibiting a particularly pronounced increase compared with both the parental lines and WT (Fig. 2). This observation aligned with the established role of GABA as a stress-responsive metabolite, which accumulates in plants as part of a conserved adaptive response to abiotic stressors (Sita and Kumar 2020; Signorelli et al. 2021).

### Upregulated expression of OsGAD1 and OsGAD3 in response to abiotic stress

**Fig. 3:**
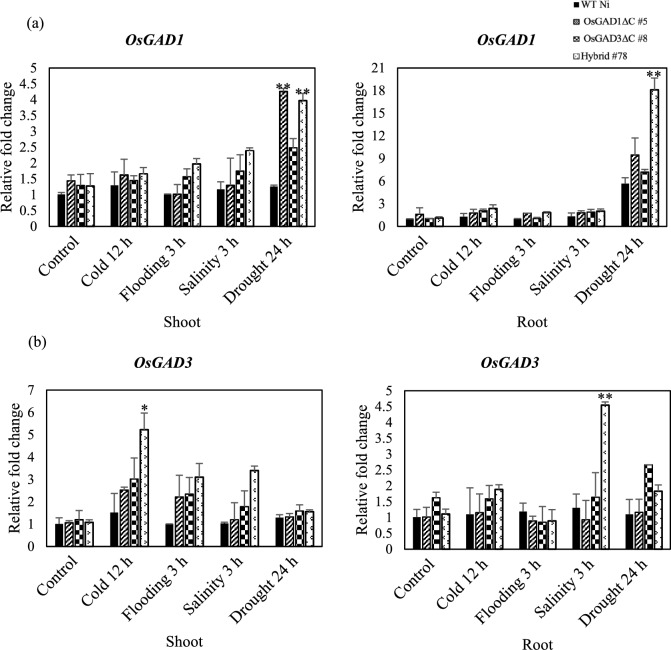
Relative expression of *OsGAD1* and *OsGAD3* genes in abiotic stress conditions. (**a**) *OsGAD1* and **(b)** *OsGAD3* gene expression in shoot and root tissues of WT-Ni, OsGAD1ΔC #5, OsGAD3ΔC #8, and Hybrid #78 in response to control (without stress treatment), cold (12 h), flooding (3 h), salinity (3 h), and drought (24 h) conditions. Bars represent the mean ± standard deviation (SD) of relative fold change. Expression levels were analyzed using the 2.^^−ΔΔCt^ method, where *TATA-binding protein (TBP2)* was used as an internal control. Statistical significance was determined by comparing the values of each rice line with WT-Ni in identical stress conditions. Asterisks denote significant differences (*P < 0.05, **P < 0.01)

RT-qPCR analysis showed significant upregulation of *OsGAD1* (Fig. 3a) and *OsGAD3* (Fig. 3b) across all four rice lines (WT Ni, OsGAD1∆C #5, OsGAD3∆C #8, and Hybrid #78) in response to abiotic stresses, particularly in drought conditions. Hybrid #78 exhibited the highest levels, with *OsGAD1* expression reaching approximately 18-fold in root tissues and *OsGAD3* nearly 12-fold in drought stress conditions. In contrast, WT Ni and OsGAD1∆C #5, displayed lower levels of upregulation. Chen et al. (2024) also reported that drought stress in rice led to increased expression of GAD, which boosted GABA levels and played a role in improving water use efficiency and enhancing stress tolerance. In addition, Wang et al. (2024) noted that in tomatoes, cold stress also stimulated GAD activity, leading to increased GABA production that aided in cellular stabilization and reduced damage from low temperatures. Conversely, in poplar trees, Ji et al. (2020) reported that among six identified GAD genes, only two showed increased expression in response to salt stress caused by NaCl. Similarly, Zhang et al. (2022) found that hypoxic conditions in tea plants elevated GABA levels through the GABA shunt pathway, resulting in the upregulation of the CsGAD1 and CsGAD2 genes.

Thus, the higher GABA accumulation observed in Hybrid #78 in stress conditions was consistent with its enhanced expression of *OsGAD1* and *OsGAD3* genes, implying a positive feedback regulation of expression of these genes by GABA accumulation.

### Differential response of free amino acid content to abiotic stress in rice vegetative tissues

The differential accumulation of amino acids in response to abiotic stress is a well-documented adaptive mechanism in plants to mitigate environmental challenges (Anzano et al. 2022; Rai 2002). The concentrations of alanine, serine, aspartic acid, glutamic acid, proline, and valine known for their roles in stress tolerance (Rossi et al. 2021), were quantified in both shoot and root tissues across four genotypes (Hybrid #78, OsGAD3∆C #8, OsGAD1∆C #5, and WT Ni) in control, cold, salt, flooding, and drought conditions. The findings highlighted the variations in amino acid accumulation in response to these stress conditions, revealing distinct differences across the different plant tissues and genotypes.

**Fig. 4:**
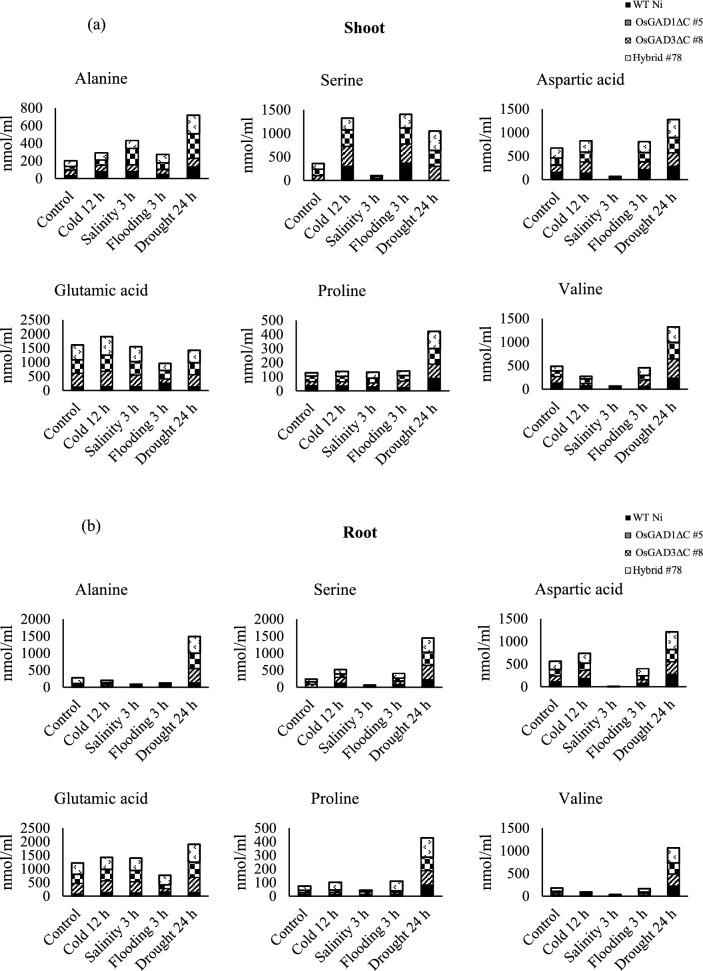
Comparative analysis of free amino acid content in shoot and root tissues after abiotic stress treatment**.** This stacked column bar graph represents the quantified levels of free amino acids: alanine, serine, aspartic acid, glutamic acid, proline, and valine, in the vegetative tissues of different rice lines subjected to abiotic stresses. Panel (**a**) illustrates the amino acid content in shoot tissues, and panel (**b**) depicts that in root tissues. WT Ni, OsGAD1ΔC #5, OsGAD3ΔC #8, and Hybrid #78 were examined in this analysis

In shoot tissues (Fig. 4a), Hybrid #78 exhibited marked increases in the levels of alanine, serine, aspartic acid, glutamic acid, proline, and valine, particularly in drought stress conditions, where the increases were most pronounced. Moderate increases were also observed in cold and salt stress conditions, whereas slight decreases were noted in flooding conditions. Similarly, OsGAD3∆C #8 displayed significant increases in drought and salt stress conditions, with smaller increases in cold stress and no significant change or slight decreases under flooding conditions. OsGAD1∆C #5 showed more modest increases during stress, with noticeable decreases in flooding conditions. WT Ni demonstrated the least responsiveness, with minor increases in amino acid levels in cold and salt stress conditions, moderate increases in drought conditions, and decreases in flooding conditions.

In root tissues (Fig. 4b), the trends largely mirrored those seen in shoot tissues, with generally higher increases in amino acid levels in drought stress conditions, especially in Hybrid #78 supporting the role of amino acid accumulation in osmotic adjustment and stress tolerance (Heinemann and Hildebrandt 2021). Decreases in amino acid levels were particularly evident in WT Ni and OsGAD1∆C #5 in flooding conditions, especially for serine, aspartic acid, and glutamic acid. These results highlighted the superior stress tolerance of Hybrid #78, which consistently showed the highest accumulation of amino acids in stress conditions. In addition, the consistently highest amino acid accumulation in Hybrid #78, particularly in drought and salt stress conditions, aligned with previous reports that linked amino acid biosynthesis to osmo-protection and stress response (Trovato et al. 2021). The data also emphasized the varying capacities of different rice lines to adapt to abiotic stress, with Hybrid #78 and OsGAD3∆C #8 showing more resilient responses compared with OsGAD1∆C #5 and WT Ni, particularly in drought and salt stress conditions. In addition, the observed decreases in amino acid levels in flooding conditions, particularly in WT Ni and OsGAD1∆C #5, suggest a disruption in amino acid metabolism during submergence, reflecting the complexity of the stress response. This aligned with findings by Komatsu et al. (2024), which demonstrated that submergence triggers significant metabolic shifts, including changes in amino acid levels, as part of the plant's adaptive stress response. The distinct responses of WT Ni and OsGAD1∆C #5 reflected the physiological variability in stress tolerance and the intricate interplay between plant metabolic pathways and environmental stressors in modulating resilience mechanisms.

### Induced abiotic stress tolerance

**Fig. 5:**
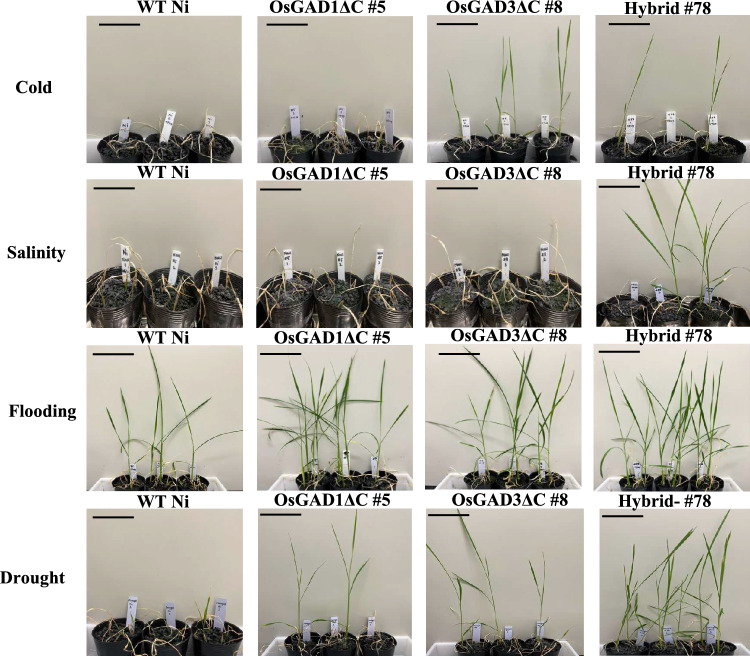
Abiotic stress tolerance in rice seedlings**.** Assessments of cold, salinity, flooding, and drought stress responses were conducted on WT Ni, OsGAD1ΔC #5, OsGAD3ΔC #8, and Hybrid #78 at the seedling stage. For cold stress evaluation, 16-day-old seedlings were subjected to 4 °C for 5 days prior to soil transplantation. Salinity stress was imposed by immersing 14-day-old seedlings in 150 mM NaCl solution for 48 h, followed by transfer to soil. Flooding stress involved immersion of seedlings in MS liquid media for 72 h, followed by transfer to soil. Drought stress was simulated by keeping seedlings on plastic plates for 6 h until approximately 65% of their initial fresh weight was lost (scale bar = 12 cm)

The survival rates of rice seedlings in various abiotic stress conditions offered key insights into the mechanisms of stress tolerance. To assess survival rates, WT Ni, OsGAD1∆C #5, OsGAD3∆C #8, and Hybrid #78 seedlings were exposed to cold, salinity, flooding, and drought stress, followed by recovery in soil (Fig. 5). Each experiment was conducted in triplicate.

In cold stress conditions (4 °C for 5 days), WT Ni and OsGAD1∆C #5 showed no survival (0%), whereas OsGAD3∆C #8 had a 33% survival rate, and Hybrid #78 showed 25% survival, indicating moderate cold tolerance (Table 1). Salinity stress (48 h) resulted in 0% survival for WT Ni, OsGAD1∆C #5, and OsGAD3∆C #8, whereas Hybrid #78 had a 33% survival rate, demonstrating strong salt tolerance. For flooding stress, WT Ni had a 33% survival rate, OsGAD1∆C #5 had 66%, OsGAD3∆C #8 had 50%, and Hybrid #78 had 83%, indicating substantial flooding tolerance. Drought stress revealed 8% survival for WT Ni, 18% for OsGAD1∆C #5, 33% for OsGAD3∆C #8, and 83% for Hybrid #78, showing strong drought tolerance.

**Table 1:** Survival rate (%) after abiotic stresses

Stress type	Survival rate (%)			
	WT Ni	OsGAD1ΔC #5	OsGAD3ΔC #8	Hybrid #78
Cold	0	0	33.33 ± 4.54^**^	25.00 ± 6.49^**^
Salinity	0	0	0	33.33 ± 4.82^**^
Flooding	33.33 ± 3.76	66.71 ± 6.21	50.31 ± 5.92	83.86 ± 4.27^*^
Drought	8.33 ± 5.02	18.00 ± 3.76	33.33 ± 7.41^*^	83.33 ± 7.84^**^

^Post^^−^^stress recovery was quantified by calculating the survival rate of seedlings after an 18^^−^^day recovery period in soil. Bars represent the mean±standard deviation (SD) (n=12 plants). Statistical significance was determined by comparing the values of each rice line with WT Ni in identical stress conditions. Asterisks denote significant differences (*^^P<0.05, **P<0.01)^

Hybrid #78 consistently exhibited the highest survival rates and vigorous growth across all stress conditions, indicating significant stress tolerance. OsGAD3∆C #8 and OsGAD1∆C #5 showed moderate to low tolerance, with WT Ni being highly sensitive to all stresses. The superior performance of Hybrid #78, especially against salt stress, suggested that hybridization enhanced stress tolerance, likely due to the complementary effects of genetic modifications in the parental lines, because the benefit of hybridization has been widely recognized in rice and other crops, where it frequently results in enhanced performance (Gu and Han 2024). The survival of Hybrid #78 and OsGAD3∆C #8 in cold stress conditions, in contrast to the non-survival of OsGAD1∆C #5, suggested that the modifications in OsGAD3∆C #8 provided a degree of cold resistance, which was further enhanced in the hybrid line. On the other hand, the difference between OsGAD1ΔC #5 and Hybrid #78, where OsGAD1ΔC #5 had higher GABA levels under control conditions, while Hybrid #78 exhibited greater survival under abiotic stress indicates that the truncation of OsGAD1 alone in OsGAD1ΔC #5 resulted in constitutively high GABA levels, which did not necessarily enhance stress resilience. Meanwhile, the co-expression of OsGAD1 and OsGAD3 truncations in Hybrid #78 facilitated controlled GABA accumulation, allowing for a more dynamic and stress-responsive adaptation rather than excessive accumulation in unstressed conditions.

We evaluated the biomass loss (Table 2), both in terms of fresh and dry weight of WT Ni, OsGAD1∆C #5, OsGAD3∆C #8, and Hybrid #78 in various abiotic stress conditions, including cold, salinity, flooding, and drought stress conditions. Hybrid #78 consistently exhibited the lowest biomass loss in all stress conditions, particularly in drought conditions, where it showed the least reduction in both fresh and dry weight. In contrast, WT Ni displayed the highest biomass loss across all conditions, indicating its greater sensitivity to stress conditions. The genome-edited lines, OsGAD1∆C #5 and OsGAD3∆C #8, demonstrated intermediate levels of biomass loss, with OsGAD3∆C #8 showing slightly better tolerance, especially in salinity and drought stress conditions. These results suggested that Hybrid #78 has superior stress tolerance, enabling it to better maintain biomass in adverse environmental conditions compared with the other genotypes.

**Table 2:** Biomass loss (%) after abiotic stresses

Stress type	Biomass loss (FW%)				Biomass loss (DW%)			
	WT Ni	OsGAD1ΔC #5	OsGAD3ΔC #8	Hybrid #78	WT Ni	OsGAD1ΔC #5	OsGAD3ΔC #8	Hybrid #78
Cold	13.54 ± 3.67	11.93 ± 1.41	6.77 ± 4.53^*^	2.33 ± 3.15^**^	14.65 ± 3.01	18.52 ± 3.89	10.32 ± 2.25	7.02 ± 3.15^*^
Salinity	17.41 ± 5.70	14.84 ± 3.68	12.67 ± 4.73	7.32 ± 3.97^**^	14.12 ± 1.43	11.96 ± 2.35	10.21 ± 1.34	5.16 ± 3.97
Flooding	22.11 ± 3.50	18.05 ± 4.69	12.87 ± 2.22^*^	6.51 ± 1.84^**^	17.53 ± 2.06	11.53 ± 1.36	8.82 ± 1.04^*^	4.97 ± 1.92^*^
Drought	69.50 ± 2.86	57.49 ± 2.9	55.95 ± 3.54	45.77 ± 3.33^*^	22.43 ± 3.77	17.26 ± 2.23	14.95 ± 1.61^*^	10.32 ± 2.29^**^

Data represented as the mean±standard deviation (SD) of fresh weight (FW) and dry weight (DW) percentage in cold, salinity, flooding, and drought stress conditions. Statistical significance was determined by comparing the values of each rice line with the wild type in identical stress conditions. Asterisks denote significant differences (*P*<0.05, **P<0.01)

The enhanced stress tolerance observed in Hybrid #78 was largely due to the increased expression of *GAD1* and *GAD3* genes, which significantly boosted GABA levels. Specifically, the superior salinity tolerance of Hybrid #78, demonstrated by its 33% survival rate in salinity stress conditions, can be attributed to it having the highest GABA content among the four rice lines. For cold stress, both Hybrid #78 and OsGAD3∆C #8 showed higher tolerance, which can be explained by the upregulation of the *GAD3* gene. The survival of Hybrid #78 and OsGAD3∆C #8 in cold conditions suggested that *GAD3* plays a crucial role in conferring cold tolerance. In the case of flooding and drought stress, Hybrid #78 exhibited the highest survival rates and the lowest biomass loss. This suggested that the combined expression of *GAD1* and *GAD3* in the hybrid resulted in an additive effect, enhancing GABA synthesis and overall stress tolerance.

Moreover, it has been reported that exogenous application of GABA can enhance tolerance to various abiotic stresses in plants, by affecting the GABA shunt, biosynthesis of secondary cell wall and plant hormones, the regulation of carbon and nitrogen metabolism, and detoxification of reactive oxygen species (Ullah et al. 2023; Chen et al. 2022). For instance, applying exogenous GABA has been found to enhance photosynthetic efficiency, increase mineral nutrients, and improve soluble sugar levels in pomegranate plants subjected to drought, salinity, and combined drought–salinity stress conditions. (Zarbakhsh and Shahsavar 2023). Exogenous GABA has also been proposed to help mitigate oxidative stress caused by higher salinity concentration by boosting antioxidant enzyme activities and lowering the levels of reactive oxygen species (Qian et al. 2024; Liu et al. 2024). Furthermore, Garg et al. (2017) reported that applying GABA to stressed plants resulted in an increase in the amounts of endogenous GABA and several other amino acids, including glutamic acid, aspartate, threonine, serine, alanine, and valine.

### Combined OsGAD1 and OsGAD3 CaMBD truncation induces DEGs in the hybrid line

**Fig. 6:**
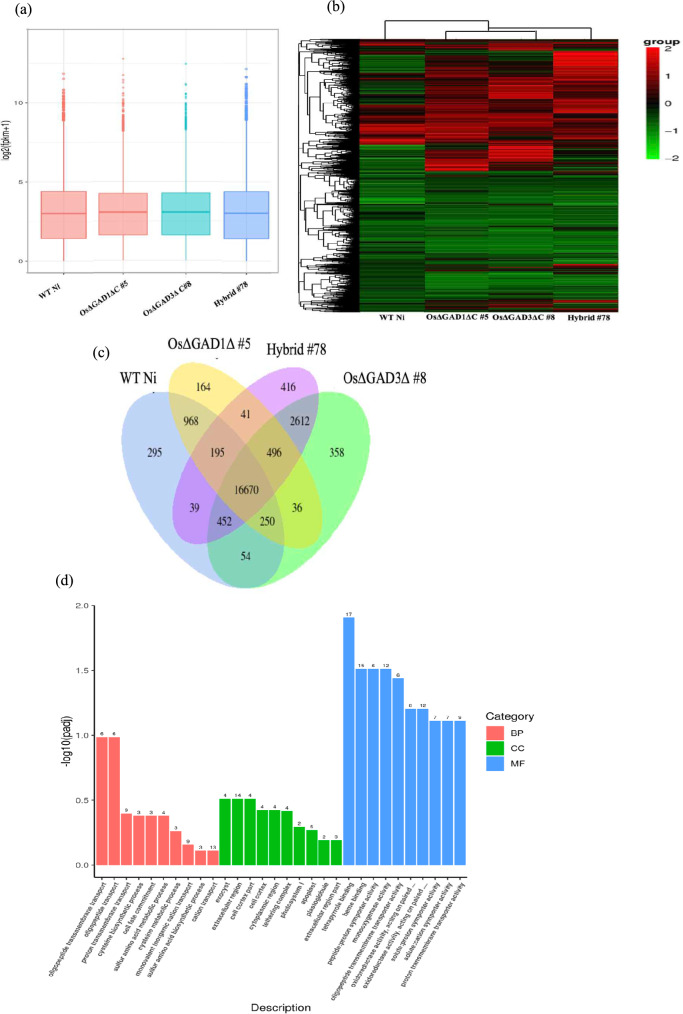
Analysis of gene expression and functional enrichment across WT Ni, OsGAD1ΔC #5, OsGAD3ΔC #8, and Hybrid #78 in control conditions. (**a**) Box plots representing the log-transformed gene expression levels (log_2_(FPKM + 1)) for four different groups: WT Ni, OsGAD1ΔC #5, OsGAD3ΔC #8, and Hybrid #78. The central line in each box represents the median expression level, the box limits represent the interquartile range (IQR), and the whiskers extend to 1.5 times the IQR. Outliers are represented by individual points. (**b**) Heatmap of differentially expressed genes (DEGs) across the four groups. The color scale ranges from red (high expression) to green (low expression). The dendrograms indicate hierarchical clustering of both genes and samples, revealing distinct expression patterns and group similarities. (**c**) Venn diagram illustrating the overlap and uniqueness of DEGs among the four groups: WT Ni, OsGAD1ΔC #5, Hybrid #78, and OsGAD3ΔC #8. Each circle represents the DEGs for one group, with numbers indicating the count of unique and shared genes. The intersections highlight common DEGs, providing insights into shared regulatory pathways and responses. (**d**) Gene ontology (GO) enrichment analysis for upregulated DEGs in Hybrid #78 vs WT Ni, categorized into biological processes (BP), cellular components (CC), and molecular functions (MF). The bar chart shows the -log_10_ (*p* value) for each GO term, with red, green, and blue bars representing BP, CC, and MF categories, respectively. Significant terms indicate key biological processes and functions affected by the genetic modifications (color figure online)

To investigate the underlying genetic factors that contribute to the enhanced stress tolerance of the Hybrid #78 line, in comparison with its parental lines OsGAD1∆C #5 and OsGAD3∆C #8, and with WT Ni, we conducted transcriptome analysis from shoot tissues in control conditions. Our findings, as depicted in Fig. 6a, showed that the median expression levels were relatively similar across all the rice lines studied. However, there was a notable difference in the interquartile range of expression levels, particularly for Hybrid #78 line. This wider range suggested greater variability in gene expression within this group. Furthermore, we observed outliers in all rice lines, with Hybrid #78 line showing the highest number and widest spread of outliers, indicating greater variability. Such variability was linked to increased adaptability and resilience to environmental stresses (Smith et al. 2015). OsGAD1ΔC #5 and OsGAD3ΔC #8 also had a notable number of outliers, though fewer than Hybrid #78. In contrast, WT Ni had few extreme values, suggesting lower deviation. Although the central tendencies of gene expression across lines were similar, the increased variability and outliers in Hybrid #78 suggested unique regulatory mechanisms or interactions specific to this line. Clustering heatmap analysis (Fig. 6b) further supported our findings regarding the genetic modifications induced by CaMBD truncation in OsGAD1ΔC #5, OsGAD3ΔC #8, Hybrid #78, and WT Ni. The heatmap clearly demonstrated that there were distinct gene expression changes in response to CaMBD truncation, particularly in Hybrid #78, compared with the parental lines and WT Ni, which was consistent with previous findings on altered gene expression levels due to specific genetic modifications (Kang et al. 2020). In addition, Venn diagram analysis revealed that there were a significant number of uniquely expressed genes present in all the rice lines, with a particularly high number in Hybrid #78 compared with the parental lines and WT Ni (Fig. 6c). This suggested that CaMBD truncation has a considerable impact on gene expression, contributing to the unique gene expression profile of Hybrid #78. Furthermore, gene ontology enrichment analysis indicated that CaMBD truncation influenced genes primarily related to molecular function (Fig. 6d). This suggested that the genetic modifications induced by CaMBD truncation resulted in alterations in the functional properties of genes, potentially affecting key molecular processes and pathways. These alterations in gene expression were associated with key molecular processes crucial for stress tolerance (Sharma et al. 2024).

### Changes in metabolic pathways and functional genes after CaMBD truncation in OsGAD1 and OsGAD3

Kyoto Encyclopedia of Genes and Genomes (KEGG) enrichment analysis identified multiple upregulated genes in Hybrid #78 compared with WT Ni in control conditions (Table S4). These genes were involved in various biological processes, including metabolism, biosynthesis, signaling, interactions, degradation, and other cellular functions, which play crucial roles in stress adaptation (the number of upregulated genes associated with each biological process is presented in Table S4). For instance, Os08g0423500, Os08g0423600, and Os08g0468700 have been reported to be involved in nitrogen metabolism, ensuring a sufficient supply of glutamate, which is crucial for the synthesis of GABA and plays a key role in stress responses (Signorelli et al. 2021; Ansari et al. 2021). In addition, Os04g0389800 participates in 2-oxocarboxylic acid metabolism (KEGG PATHWAY: Ko01210, https://www.genome.jp/dbget-bin/www_bget?ko01210), generating intermediates for the GABA shunt, linking carbon and nitrogen metabolism to enhance GABA production. Os11g0210600 is associated with gluconeogenesis, providing TCA cycle precursors that support GABA synthesis, whereas Os10g0465700 is involved in starch and sucrose metabolism, promoting glycolysis and subsequent GABA production (Chen et al. 2020). These genes collectively enhance GABA synthesis, contributing to the high stress tolerance observed in Hybrid #78.

On the other hand, transcript analysis showed several stress-related genes to be upregulated in OsGAD1∆C #5, OsGAD3∆C #8, and Hybrid #78 lines compared with WT Ni (Fig. S6) in control conditions. The expression of *OsNAC3*, a gene involved in drought and salinity stress responses, varied across the rice lines, with Hybrid #78 showing the highest levels, indicating a strong response to these stresses. OsGAD3ΔC #8 had moderate expression, suggesting intermediate adaptation to drought and salinity, whereas WT Ni and OsGAD1ΔC #5 exhibited lower *OsNAC3* expression, indicating a less pronounced response. This suggested that Hybrid #78 may have an enhanced ability to cope with drought and salinity stress compared with the other lines. *OsDST* expression was markedly higher in Hybrid #78 compared with OsGAD1∆C #5, OsGAD3∆C #8, and WT Ni. Given that *OsDST* is associated with drought and salinity tolerance, this upregulation indicated that Hybrid #78 may possess improved mechanisms for tolerating these specific stress conditions. The expression of *OsSGL* was elevated in Hybrid #78 and OsGAD3ΔC #8 compared with WT Ni and OsGAD1ΔC #5. *OsSGL* is linked to sugar metabolism and stress responses, indicating that these genotypes, particularly Hybrid #78, may have enhanced metabolic adjustments contributing to stress resilience. *HSP70* had its highest expression level in Hybrid #78. This gene encodes a heat shock protein that assists in protein folding and protection in stress conditions (Kumar et al. 2024), suggesting that Hybrid #78 may have an enhanced capacity to manage protein damage during stress.

In addition, the RT-qPCR analysis revealed upregulation of the expression levels of various stress-related genes across the genome-edited lines (OsGAD1∆C #5 and OsGAD3∆C #8) and, most notably, in Hybrid #78, compared with WT Ni. Specifically, previously reported abiotic stress-associated genes such as cold stress (*OsADC1, OsTAF2, OsSAP1)* (Fig. S7) (Peremarti et al. 2010; Kothari et al. 2016), flooding stress (*OsGolS1, OsERF68, OsRAB16A*) (Fig. S8) (Martins et al. 2022; Haque et al. 2023; García et al. 2024), salinity stress (*OsMYB30, OsHAK5, OsNAC3*) (Fig. S9) (Zhang et al. 2021; Hussain et al. 2024), and drought stress (*OsDREB2B, OsDST, OsHSF13*) (Fig. S10) (Matsukura et al. 2010; Santosh Kumar et al. 2020; Sirohi et al. 2020) exhibited increased expression levels. This suggests that the truncation of the inhibitory CaMBD domain from OsGAD1 and OsGAD3 not only enhanced the expression of these genes but also potentially primed the genome-edited lines and Hybrid #78 for improved stress tolerance. Similarly, Galon et al. (2008) demonstrated that calmodulin-binding transcription activator 3 (CAMTA3) in *Arabidopsis* regulates the expression of genes associated with biotic defense responses. On the other hand, the significantly upregulated expression of *OsHAK5* (Fig. S9) in Hybrid #78 suggests a stronger CBL1/CBL9–CIPK23 regulatory mechanism, which enhances K⁺ homeostasis and water retention under salinity and drought stress (Ródenas and Vert 2021). However, the elevated transcript levels of the stress-related genes may indicate an activation of pre-defense mechanisms, equipping the genome-edited and hybrid lines with a more effective response to environmental stressors compared with WT Ni. This enhanced pre-defense state likely contributes to the superior resilience observed in Hybrid line #78 in various stress conditions.

In conclusion, the simultaneous upregulation of *OsGAD1*, *OsGAD3*, metabolic pathways, and stress-responsive genes in the genome-edited lines and Hybrid #78 demonstrated a genetic preconditioning strategy that ensures a consistent supply of essential metabolites, such as GABA, which is critical for stress tolerance. This preconditioning allows these plants to quickly activate defense mechanisms, reducing damage and maintaining physiological functions in stress conditions. In OsGAD1ΔC #5, OsGAD3ΔC #8, and Hybrid #78, this creates a strong network of stress tolerance, with enhanced metabolic capacity supporting GABA production and upregulated stress-related genes ensuring a rapid response to stressors. Furthermore, this study established a foundation for future research into the molecular mechanisms underlying stress adaptation, with a particular focus on the role of GABA as a key metabolite in these processes. By elucidating how GABA synthesis and signaling are regulated in abiotic stress conditions through *OsGAD1* and *OsGAD3* genes, these findings provide valuable insights for enhancing stress tolerance in rice. Expanding this approach to other crops and stress conditions may advance our understanding of abiotic stress mechanisms, supporting efforts to sustain agricultural productivity in response to changing environmental conditions.

## Supplementary Information

Below is the link to the electronic supplementary material.

Supplementary file1 (DOCX 4750 KB)

## Data Availability

The data and materials supporting the findings of this study can be provided from the corresponding author upon request.
