# Supplementary material for: Double-truncated version of OsGADs leads to higher GABA accumulation and stronger stress tolerance in Oryza sativa L. var. japonica
Source: Plant Cell Rep. 2025 Apr 8;44(5):95. doi: 10.1007/s00299-025-03477-y (PMC11978549; doi:10.1007/s00299-025-03477-y)
Supplement: Supplementary file 1 — Supplementary file1 (DOCX 4750 KB) [file 299_2025_3477_MOESM1_ESM.docx]

**Plant Cell Reports**

**Double Truncated Version of OsGADs Leads to Higher GABA Accumulation and Stronger Stress Tolerance in Oryza sativa L. var. *japonica***

**Ummey Kulsum^1^, Nadia Akter^1,2^, Kazuhito Akama^1*^**

^1^ Graduate School of Natural Science and Technology, Shimane University, 1060, Nishikawatsu, Matsue, Shimane 690-8504, Japan

^2^ *Present address:* Genetic Resources and Seed Division, Bangladesh Rice Research Institute, Gazipur 1701, Bangladesh

***Corresponding author:** [akama@life.shimane-u.ac.jp](mailto:akama@life.shimane-u.ac.jp) (Kazuhito Akama)

**Table S1.** Grain weight and relative GABA content in the WT Ni and OsGAD1 genome-edited lines

| **Rice lines** | **Brown rice (mg/grain)** | **GABA (fold change)** |
| --- | --- | --- |
| WT Ni | 18.8 | 1 |
| OsGAD1ΔC #5 | 20.8 | 6.6 |
| OsGAD1ΔC #206 | 20.7 | 1.1 |
| OsGAD1ΔC #210 | 20.3 | 2.7 |
| OsGAD1ΔC #219 | 18.7 | 2 |

**Table S2.** Agronomic traits of WT Ni, OsGAD1ΔC #5, OsGAD3ΔC #8, and Hybrid #78.

| **Rice line** | **Dry weight (g)** | **No. of branches** | **Leaf blade size (cm)** | **No of panicles** | **Panicle length (cm)** | **Clum length (cm)** | **No of seeds per panicle** | **Weight of 1000 seeds (g)** | **Total weight of seeds (g)** | **Ripening rate (%)** |
| --- | --- | --- | --- | --- | --- | --- | --- | --- | --- | --- |
| WT Ni | 175 ± 7.0 | 40.5 ± 6.0 | 64.36 ± 5.2 | 37.33 ± 3.9 | 20.03 ± 2.5 | 68.9 ± 5.1 | 119.9 ± 8.5 | 26.8 ± 1.5 | 125.9 ± 4.9 | 92.51 ± 2.9 |
| OsGAD1ΔC #5 | 139.6 ± 19.9 | 36.3 ± 6.0 | 60.0 ± 2.9 | 35.4 ± 4.0 | 19.5 ± 1.4 | 70.4 ± 1.6 | 105.3 ± 10.4 | 25.7 ± 0.9 | 101.2 ± 4.7 | 91.8 ± 3.0 |
| OsGAD3ΔC #8 | 161.2 ± 4.1 | 32.57 ± 6.7 | 69.65 ± 2.5 | 28.71 ± 7.1 | 20.91 ± 1.2 | 87 ± 6.2** | 111.3 ± 8.2 | 22.8 ±1.2 | 75.34 ± 7.2 | 81.01 ± 4.4 |
| Hybrid #78 | 154.42 ± 8.4 | 33.14 ± 5.1 | 68.65 ± 6.6 | 26.42 ± 4.8 | 23.28 ± 1.8* | 81.82 ± 4.6* | 105.93 ± 5.5 | 27.09 ± 1.4 | 86.52 ± 6.5 | 90.76 ± 2.1 |

^Data represented as mean ± standard deviation (n=5). Asterisks indicate significant differences (^*^*P^*^<0.05^*^, **P^*^<0.01). Significant differences were determined using Student’s^ *^t^*^-test by comparing the genome-edited lines with wild-type Ni.^

**Table S3.** GABA contents of vegetative tissues from WT Ni and genome-edited plants

| **Rice lines** | **(GABA nmol/mg)** | | |
| --- | --- | --- | --- |
|  | **Leaf** | **Stem** | **Root** |
| WT Ni | 37.87 ± 1.77 | 12.82 ± 1.73 | 49.85 ± 1.20 |
| OsGAD1ΔC #5 | 62.32 ± 6.10* | 28.49 ± 2.29* | 78.87 ± 1.14* |
| OsGAD3ΔC #8 | 59.51 ± 3.28* | 25.56 ± 1.56* | 81.56 ± 6.51* |
| Hybrid #78 | 79.64 ± 2.88* | 32.73 ± 1.82** | 104.40 ± 2.61** |

^Data represented as mean ± standard deviation (n=3). Asterisks indicate significant differences (^*^*P^*^<0.05^*^, **P^*^<0.01). Significant differences were determined using Student’s^ *^t^*^-test by comparing the genome-edited lines with wild-type Ni.^

**Table S4.** Descriptions of upregulated genes associated with KEGG pathways in Hybrid #78 compared with WT Ni in control conditions.

|  | **KEGG ID** | **Description** | **Gene ID** | **Number of genes** | **Kegg ID** |
| --- | --- | --- | --- | --- | --- |
| Metabolism | dosa00910 | Nitrogen metabolism | Os08g0423500/Os08g0423600/Os08g0468700 | 3 | dosa:Os08t0423500-00/dosa:Os08t0423600-00/dosa:Os08t0468700-00 |
|  | dosa01212 | Fatty acid metabolism | Os04g0116600 | 3 | dosa:Os04t0116600-01 |
|  | dosa00270 | Cysteine and methionine metabolism | Os03g0196600/Os06g0564700/Os06g0175800/Os07g0689600 | 4 | dosa:Os03t0196600-01/dosa:Os06t0564700-01/dosa:Os06t0175800-01/dosa:Os07t0689600-01 |
|  | dosa00052 | Galactose metabolism | Os07g0687900/Os07g0209100 | 2 | dosa:Os07t0687900-01/dosa:Os07t0209100-01 |
|  | dosa00053 | Ascorbate and aldarate metabolism | Os04g0361500/Os04g0360500 | 2 | dosa:Os04t0361500-00/dosa:Os04t0360500-01 |
|  | dosa00450 | Seleno compound metabolism | Os06g0175800 | 1 | dosa:Os06t0175800-01 |
|  | dosa00750 | Vitamin B6 metabolism | Os02g0226200 | 1 | dosa:Os02t0226200-01 |
|  | dosa00650 | Butanoate metabolism | Os04g0389800 | 1 | dosa:Os04t0389800-01 |
|  | dosa00350 | Tyrosine metabolism | Os11g0210600 | 1 | dosa:Os11t0210600-01 |
|  | dosa00592 | alpha-Linolenic acid metabolism | Os03g0225900 | 1 | dosa:Os03t0225900-01 |
|  | dosa01210 | 2-Oxocarboxylic acid metabolism | Os04g0389800 | 1 | dosa:Os04t0389800-01 |
|  | dosa00051 | Fructose and mannose metabolism | Os03g0828300 | 1 | dosa:Os03t0828500-01 |
|  | dosa00240 | Pyrimidine metabolism | Os12g0123500 | 1 | dosa:Os12t0123500-01 |
|  | dosa00630 | Glyoxylate and dicarboxylate metabolism | Os07g0529000 | 1 | dosa:Os07t0529000-01 |
|  | dosa00564 | Glycerophospholipid metabolism | Os01g0329000 | 1 | dosa:Os01t0329000-01 |
|  | dosa00230 | Purine metabolism | Os12g0123500 | 1 | dosa:Os12t0123500-01 |
|  | dosa00620 | Pyruvate metabolism | Os11g0210600 | 1 | dosa:Os11t0210600-01 |
|  | dosa00561 | Glycerolipid metabolism | Os01g0329000 | 1 | dosa:Os01t0329000-01 |
|  | dosa00010 | Glycolysis / gluconeogenesis | Os11g0210600 | 1 | dosa:Os11t0210600-01 |
|  | dosa00500 | Starch and sucrose metabolism | Os10g0465700 | 1 | dosa:Os10t0465700-01 |
|  | dosa00920 | Sulfur metabolism | Os03g0196600/Os06g0564700 | 2 | dosa:Os03t0196600-01/dosa:Os06t0564700-01 |
|  | dosa00380 | Tryptophan metabolism | Os09g0344500 | 1 | dosa:Os09t0344500-01 |
|  | dosa01232 | Nucleotide metabolism | Os12g0123500 | 1 | dosa:Os12t0123500-01 |
|  | dosa00480 | Glutathione metabolism | Os10g0527800 | 1 | dosa:Os10t0527800-01 |
|  | dosa00500 | Starch and sucrose metabolism | Os10g0465700 | 1 | dosa:Os10t0465700-01 |
| Biosynthesis | dosa01230 | Biosynthesis of amino acids | Os03g0196600/Os06g0564700/Os06g0175800/Os04g0389800 | 4 | dosa:Os03t0196600-01/dosa:Os06t0564700-01/dosa:Os06t0175800-01/dosa:Os04t0389800-01 |
|  | dosa00999 | Biosynthesis of various plant secondary metabolites | Os02g0306401/Os07g0689600/Os04g0167800 | 3 | dosa:Os02t0306401-00/dosa:Os07t0689600-01/dosa:Os04t0167800-01 |
|  | dosa00290 | Valine, leucine, and isoleucine biosynthesis | Os04g0389800 | 1 | dosa:Os04t0389800-01 |
|  | dosa00908 | Zeatin biosynthesis | Os10g0178500 | 1 | dosa:Os10t0178500-01 |
|  | dosa00941 | Flavonoid biosynthesis | Os10g0317900 | 1 | dosa:Os10t0317900-01 |
|  | dosa01040 | Biosynthesis of unsaturated fatty acids | Os04g0116600 | 1 | dosa:Os04t0116600-01 |
|  | dosa00073 | Cutin, suberin, and wax biosynthesis | Os04g0354600 | 1 | dosa:Os04t0354600-01 |
|  | dosa00904 | Diterpenoid biosynthesis | Os03g0856700 | 1 | dosa:Os03t0856700-01 |
|  | dosa00130 | Ubiquinone and other terpenoid-quinone biosynthesis | Os08g0143300 | 1 | dosa:Os08t0143300-00 |
|  | dosa00770 | Pantothenate and CoA biosynthesis | Os04g0389800 | 1 | dosa:Os04t0389800-01 |
|  | dosa00940 | Phenylpropanoid biosynthesis | Os04g0689000/Os10g0512400/Os03g0339300/Os08g0143300 | 4 | dosa:Os04t0689000-01/dosa:Os10t0512400-01/dosa:Os07t0676900-01/dosa:Os08t0143300-00 |
|  | dosa01240 | Biosynthesis of cofactors | Os04g0361500/Os02g0226200/Os04g0360500 | 3 | dosa:Os04t0361500-00/dosa:Os02t0226200-01/dosa:Os04t0360500-01 |
| Signalling and interaction | dosa00196 | Photosynthesis - antenna proteins | Os01g0600900/Os01g0720500 | 2 | dosa:Os01t0600900-02/dosa:Os01t0720500-01 |
|  | dosa04626 | Plant-pathogen interaction | Os05g0380900/Os01g0955100/Os10g0191300/Os05g0343400/Os03g0382100/Os06g0262800 | 6 | dosa:Os05t0380900-01/dosa:Os01t0955100-01/dosa:Os10t0191300-01/dosa:Os05t0343400-01/dosa:Os03t0382100-01/dosa:Os06t0262800-01 |
|  | dosa04016 | MAPK signaling pathway - plant | Os10g0191300/Os05g0343400/Os09g0325700 | 3 | dosa:Os10t0191300-01/dosa:Os05t0343400-01/dosa:Os09t0325700-01 |
|  | dosa04075 | Plant hormone signal transduction | Os02g0643800/Os10g0191300/Os02g0769100/Os09g0325700 | 4 | dosa:Os02t0643800-01/dosa:Os10t0191300-01/dosa:Os02t0769100-01/dosa:Os09t0325700-01 |
| Degradation/Utilization | dosa00062 | Fatty acid elongation | Os04g0116600/Os03g0382100/Os06g0262800 | 3 | dosa:Os04t0116600-01/dosa:Os03t0382100-01/dosa:Os06t0262800-01 |
|  | dosa04122 | Sulfur relay system | Os01g0598900 | 1 | dosa:Os01t0598900-00 |
|  | dosa00071 | Fatty acid degradation | Os11g0210600 | 1 | dosa:Os11t0210600-01 |
|  | dosa03018 | RNA degradation | Os04g0684900 | 1 | dosa:Os04t0684900-01 |
|  | dosa04120 | Ubiquitin-mediated proteolysis | Os01g0124900 | 1 | dosa:Os01t0124900-00 |
| Other cellular process | dosa04146 | Peroxisome | Os04g0354600 | 1 | dosa:Os04t0354600-01 |
|  | dosa03010 | Ribosome | Os07g0565100 | 1 | dosa:Os04t0613600-00 |

**Table S5**. Primer list used in this study

| **Primer** | **Sequence (5’-3’)** | **Purpose** |
| --- | --- | --- |
| OsGAD1gRNA F1 | GTTGGCCCGATTGCTGCTTCGCGA | Genome editing |
| OsGAD1gRNA R1 | AAACTCGCGAAGCAGCAATCGGGC |  |
| OsGAD1gRNA F2 | GTTGGCAATCGGGCGACGATGGCG |  |
| OsGAD1-329 F | TCGTCATCAGGGAGGACTTC | Confirmation of genome editing |
| OsGAD1-329 R | CGTACACCGCCAGTCAGTC |  |
| OsGAD3-F57 | GTCCTCGACATCGAGAAGGT |  |
| OsGAD3-R379 | AGAATCGAAGGCTCCACTCA |  |
| OsHSP70-F | ACCGTCTTTGATGCCAAG | RT-qPCR analysis |
| OsHSP70-R | CTCAGCAATCTCACGCAT |  |
| OsNAC3-F | GAAGAACGAGTGGGAGAAGATG |  |
| OsNAC3-R | GCGAGCATGGAGAGGTC |  |
| OsMYB30-F | GTGGATCAACTACCTCCGC |  |
| OsMYB30-R | TTCTTGATCTCGTTGTCCGTC |  |
| OsERF68-F | TCATCTACGACTACATCCCGG |  |
| OsERF68-R | GTTCTTCCGCTCCCTCTTC |  |
| OsHAK5-F | CCAAAGCCATACAGCCAAG |  |
| OsHAK5-R | TCCTTGATCCCGTTGGTAAAG |  |
| OsRAB16A-F | GCTCAAGCTCGTCTGAGG |  |
| OsRAB16A-R | GTGTCGGTGGTGGTGGTG |  |
| OsTAF2-F | CTTGCTTTACCAGGTCTTAAGC |  |
| OsTAF2-R | GACACTGTGGAAAAATGAGATG |  |
| OsADC1-F | TCCCGATCATCCCAATCCAG |  |
| OsADC1-R | GAGGAACATGCCGAGGTAGT |  |
| OsSGL-F | CACAGCAGAAGAAGCAGAGC |  |
| OsSGL-R | CTAATAGGCGGTGTGGTGTTG |  |
| OsSAP1-F | CGCGACAAGAAGGATCAGGA |  |
| OsSAP1-R | GGTGACGACAAAGAAGACGG |  |
| OsGolS1-F | TGTGCAGCGGGTTCGAAG |  |
| OsGolS1-R | GGAAGTACTTGACGGCGC |  |
| OsDST-F | AAGTTCTTGAAGTCGCAGGC |  |
| OsDST-R | CCCCAACGCCAGCAGTAG |  |
| OsDSR-1 F | CAGATTCATGGGTTATGG |  |
| OsDSR-1 R | GACAGCAGCTTCTTGATA |  |
| OsHSF13-F | AACACCTACGGATTTAGGAAAG |  |
| OsHSF13-R | CTCAATCTCTTCTTCCATCC |  |
| OsDREB2B-F | GTGGAGGCGAGGAAAGTACTGGA |  |
| OsDREB2B-R | CCTGTGGATCAAGCTCCTGC |  |
| OsGAD1-F | ATGGGACTGACTGGCGGTGTA |  |
| OsGAD1-R | AGGAGGAAGGAGATTGGCAAGC |  |
| OsGAD2-F | AACCAAGGGCGTTTGCTAGAC |  |
| OsGAD2-R | AAGAAGGTTTAGTACGCTCCCA |  |
| OsGAD3-F | TCCACAAATCAAGACGCTGCTG |  |
| OsGAD3-R | GGACCTAGAATCGAAGGCTCCA |  |
| OsGAD4-F | ACCGTCTCAAGTCTGCTCTCAT |  |
| OsGAD4-R | TCAATTCACTGCTACACACCCA |  |
| TBP2-F | TGGTCTGGAGGAGCGTATAGCA | Internal control for RT-qPCR |
| TBP2-R | CAAGTCTCTCAGTCACCCAAGC |  |

5256 5315

TCCAGCGCCATCGCGAAGCAGCAATCGGGCGACGATGGCGTGGTCACCAAGAAGAGCGTC

S S A I A K Q Q S G D D G V V T K K S V

5316 5375

CTGGAGACCGAGAGGGAGATCTTCGCGTACTGGAGGGACCAGGTGAAGAAGAAGCAGACC

L E T E R E I F A Y W R D Q V K K K Q T

5376 5435

GGAATCTGCTAGTGTGGCTCTGTGAGAAATGCTTGAATAACGTGGCATGCTCGATTTGTG

G I C * C G S V R N A * I T W H A R F V

**↓**

**↓**

**↓**

**F1**

**F2**

**R1**

**(a)**


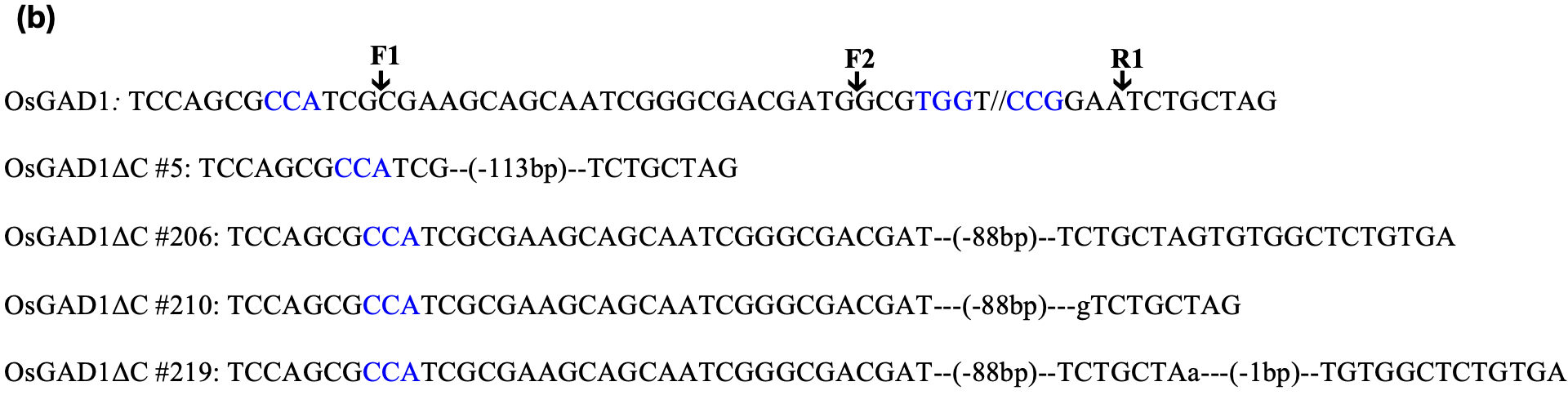


OsGAD1-SSAIAKQQSGDDGVVTKKSVLETEREIFAYWRDQVKKKQTGIC

OsGAD1ΔC #5: SSAI*VC*

OsGAD1ΔC #206: SSAIAKQQSGDD*SASVAL*

OsGAD1ΔC #210: SSAIAKQQSGDD*VC*

OsGAD1ΔC #219: SSAIAKQQSGDD*SANVAL*

**(c)**

**Fig. S1 CRISPR/Cas9-mediated production of CaMBD-truncated OsGAD1 genome-edited plants.** (a) The nucleotide and corresponding amino acid sequences of OsGAD1 (AB056060) are shown. The CaMBD region is underlined. The guide RNA (gRNA) sequence used for editing is highlighted in red, with the complementary sequence of the protospacer adjacent motif (PAM) shown in blue. F1, F2, and R1 mark the targeted cleavage sites within the gene. (b) The nucleotide sequence of wild-type *OsGAD1* and the resulting genome-edited sequences. The dashed line indicates the deletion (bp= base pair), and lowercase letters indicate insertion introduced as a result of the genome editing process. (c)The amino acid sequences of the resulting genome-edited sequences are shown. The wild-type OsGAD1 (Ni) sequence includes the CaMBD region, highlighted in red. OsGAD1ΔC refers to four genome-edited lines with truncated CaMBD regions. The sequence highlighted in teal represents the additional amino acids.


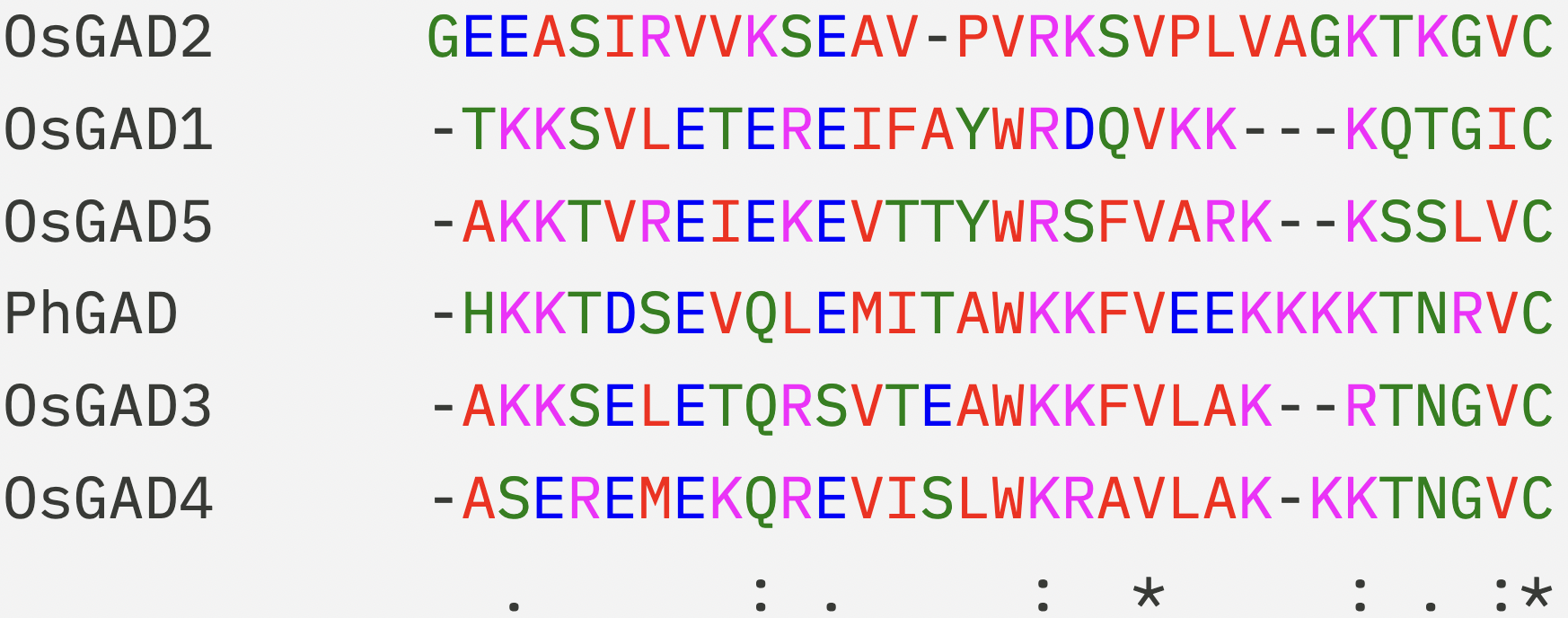


(a)


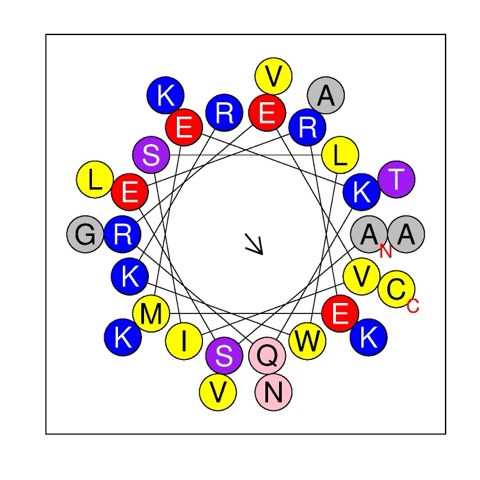

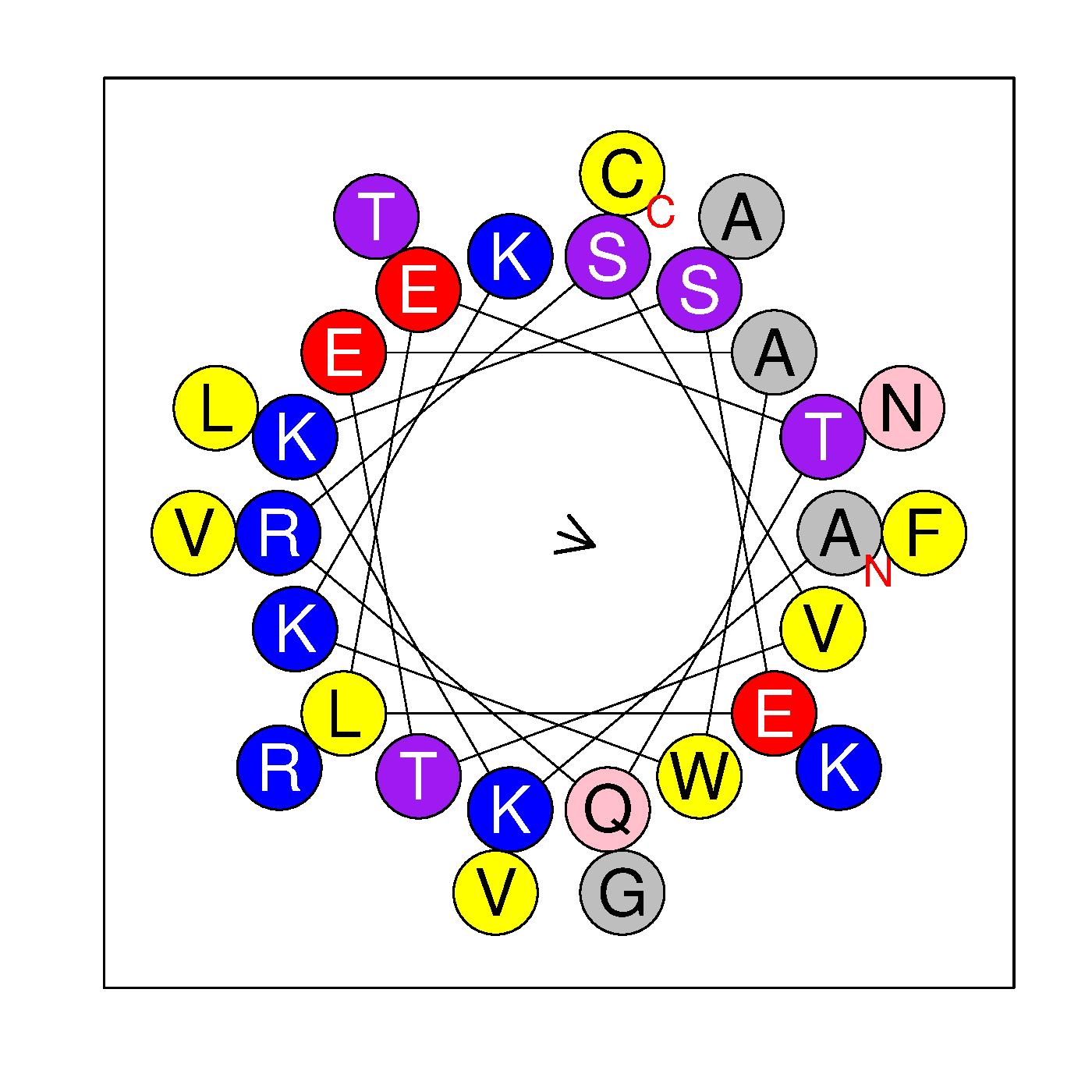

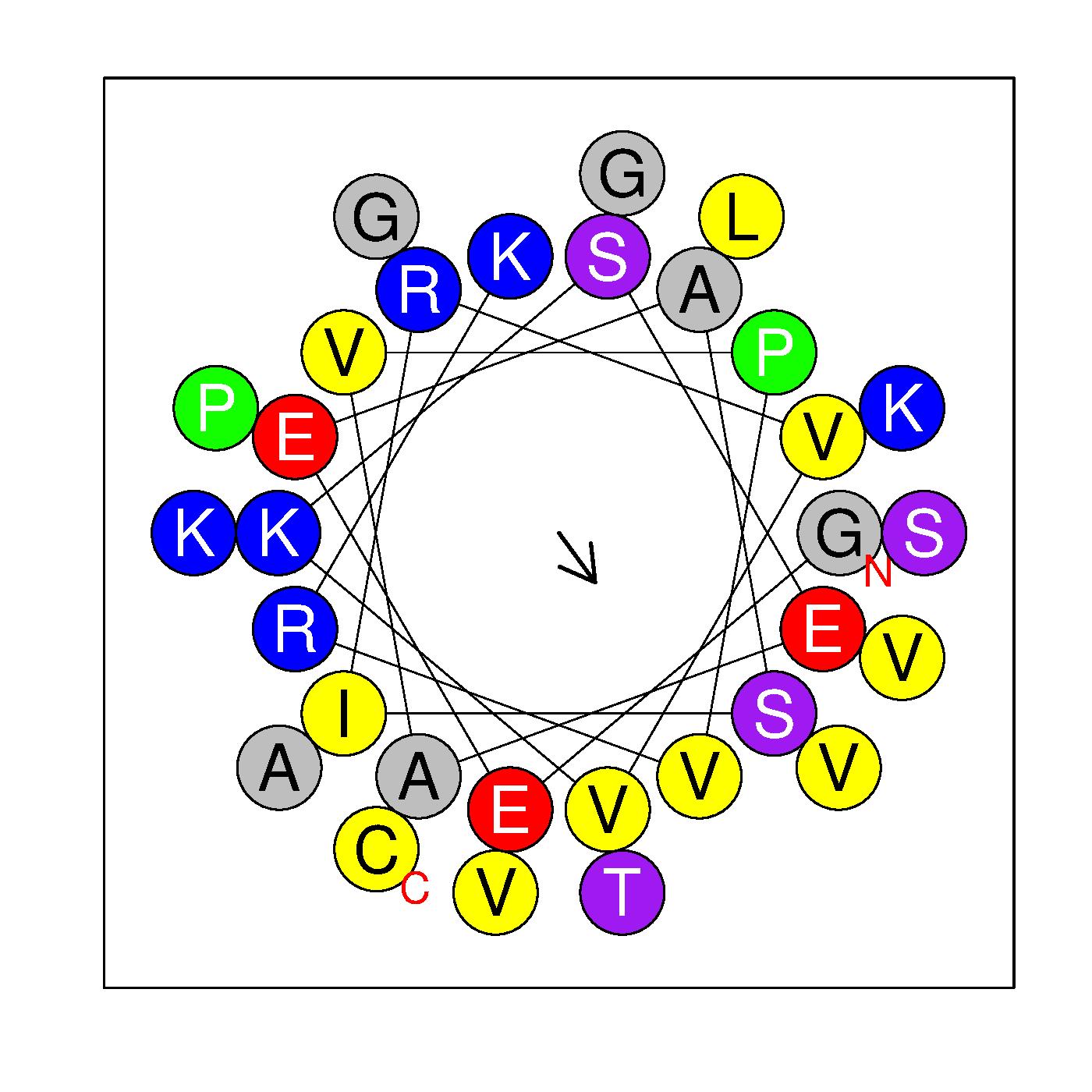

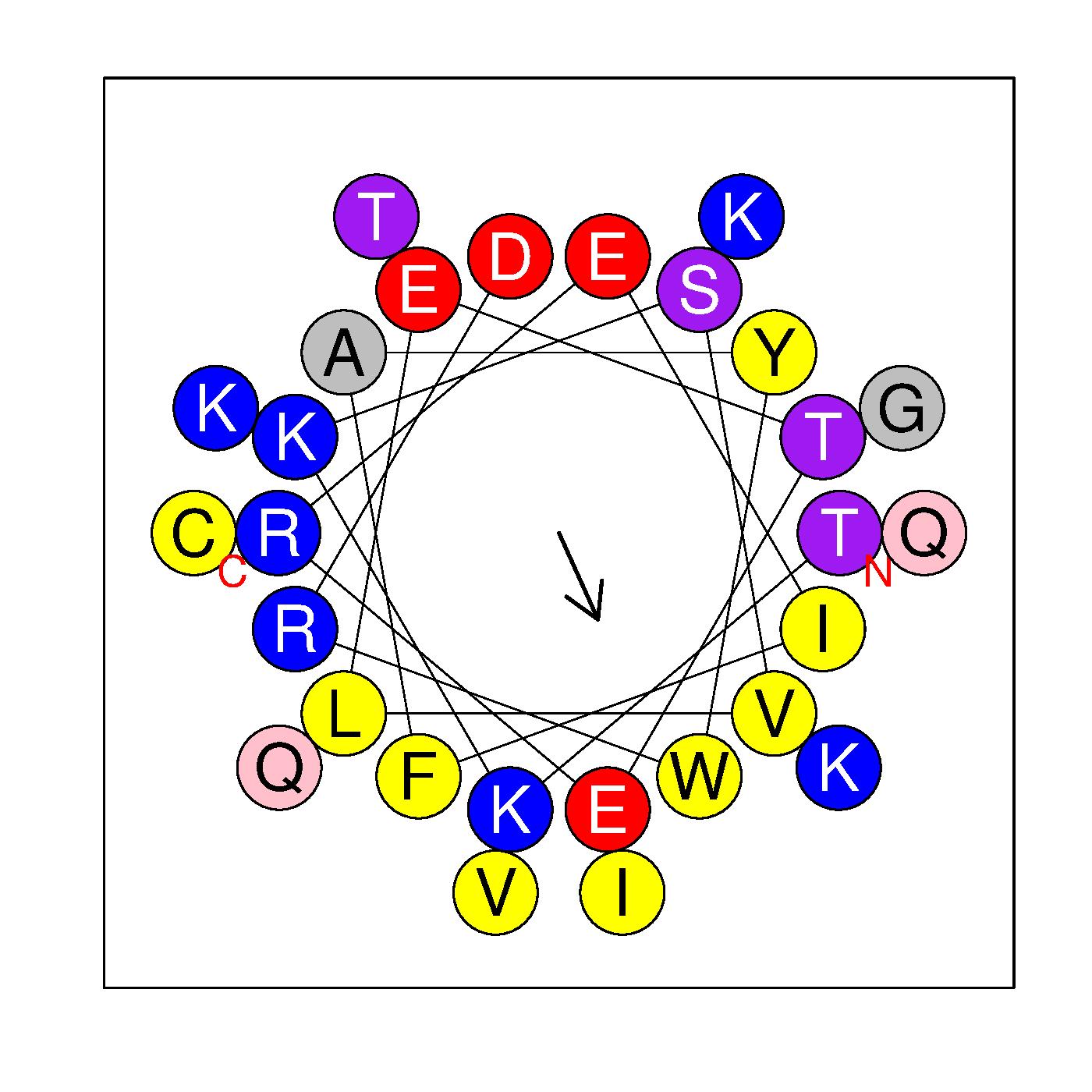


OsGAD3

OsGAD4

OsGAD2

OsGAD1

(b)

**Fig. S2 Comparison of the C-terminal regions of plant glutamate decarboxylases (GADs).** (a) Multiple sequence alignment of the C-terminal regions from *Oryza sativa* (*Os*) and *Petunia hybrida* (*Ph*) GADs using Clustal Omega (<https://www.ebi.ac.uk/jdispatcher/>). Key residues critical for calmodulin (CaM) binding, tryptophan (W) and lysine (K), are denoted using a dot and a bold line, respectively. Pseudosubstrate residues E476 and E480 in *PhGAD*, as reported by Arazi et al. (1995), are indicated with stars. The analyzed sequences include *Os*GAD1 (AB056060), *Os*GAD2 (AB056061), *Os*GAD3 (AK071556), *Os*GAD4 (AK101171), *Os*GAD5 (AK070858), and *Ph*GAD (L16977). (b) α-helical wheel projection of amino acid residues displayed using HeliQuest (<https://heliquest.ipmc.cnrs.fr/index.html>). The α-helical wheel diagram represents the amphipathic helical structure of the calmodulin-binding domain (CaMBD) of four OsGADs. Amino acid residues are displayed as colored circles, arranged to show their spatial orientation within the helix. Hydrophobic residues (yellow) cluster on one side, forming a hydrophobic face, whereas hydrophilic residues, including positively charged (blue), negatively charged (red), polar (purple), and special residues (pink and gray), form the opposite face. The arrow in the middle indicates the direction of the hydrophobic face, which interacts with hydrophobic binding pocket of calmodulin in a calcium-dependent manner. Tryptophan (W) (green arrow) plays a key role in anchoring the helix to calmodulin via strong hydrophobic interactions, and lysine (K) (blue arrow) contributes to electrostatic interactions with negatively charged residues of calmodulin, stabilizing the binding. Glutamic acid (E) (red arrow) residues act as pseudosubstrates. This amphipathic arrangement is essential for the function of CaMBD in facilitating GAD activity in response to calcium signaling. Lines connecting residues highlight spatial proximity and potential interactions within the helix.


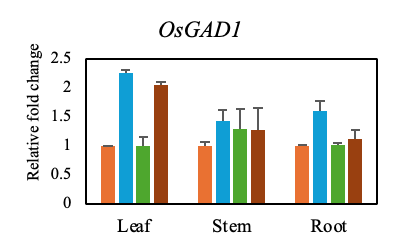

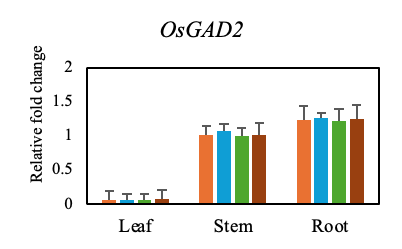

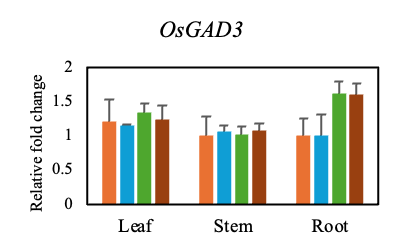

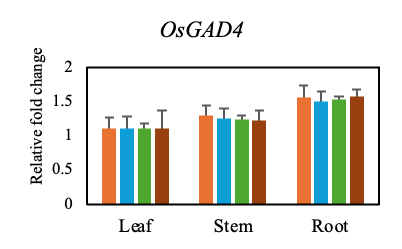

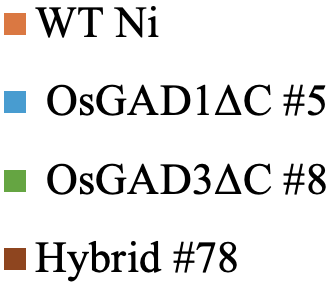


(a)

(d)

(c)

(b)

*

*

**

**

*

**Fig. S3 Relative expression of four *OsGADs* in different vegetative tissues of the rice seedlings.** Expression of (a) *OsGAD1* (AB056060), (b) *OsGAD2* (AB056061), (c) *OsGAD3* (AK071556) and (d) *OsGAD4* (AK101171) in leaf, stem, and root tissues of WT Ni, OsGAD1ΔC #5, OsGAD3ΔC #8, and Hybrid #78. Bars represent the mean ± standard deviation (SD) (n=3) of relative fold change. Expression levels were analyzed using the 2^^-ΔΔCt^ method, where TATA-binding protein (TBP2) was used as an internal control. Statistical significance was assessed by comparing the values to those of the wild-type. Asterisks denote significant differences (**P*<0.05, ***P*<0.01)*.*


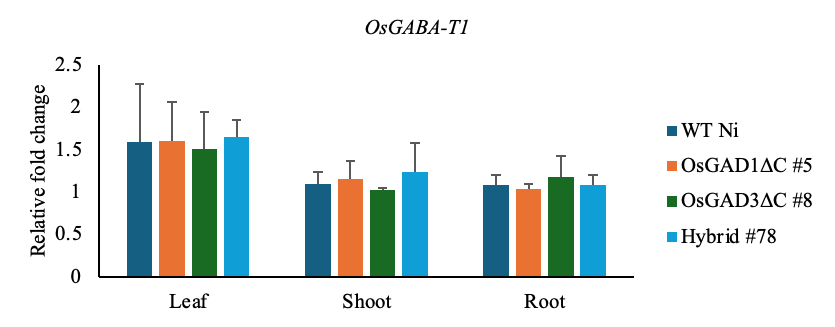

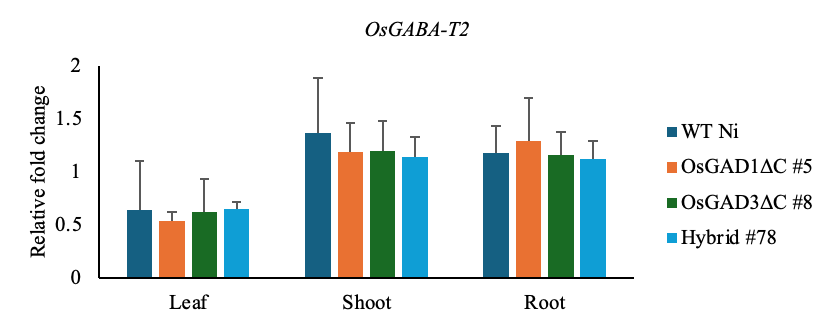

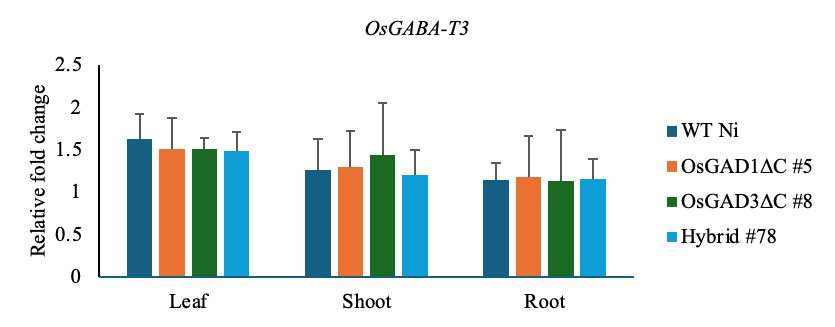


**Fig. S4 Relative expression of three *OsGABA-T* in different vegetative tissues of the rice seedlings.** Expression of *OsGABA-T1*, *OsGABA-T2*, and *OsGABA-T3* in leaf, stem, and root tissue of WT Ni, OsGAD1ΔC #5, OsGAD3ΔC #8, and Hybrid #78. Bars represent the mean ± standard deviation (SD) (n=3) of relative fold change. Expression levels were analyzed using the 2^^-ΔΔCt^ method, where TATA-binding protein (TBP2) was used as an internal control.

(a)

(b)


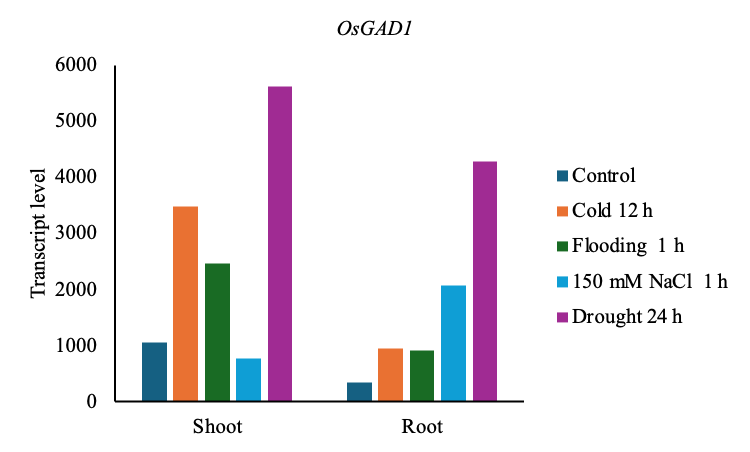

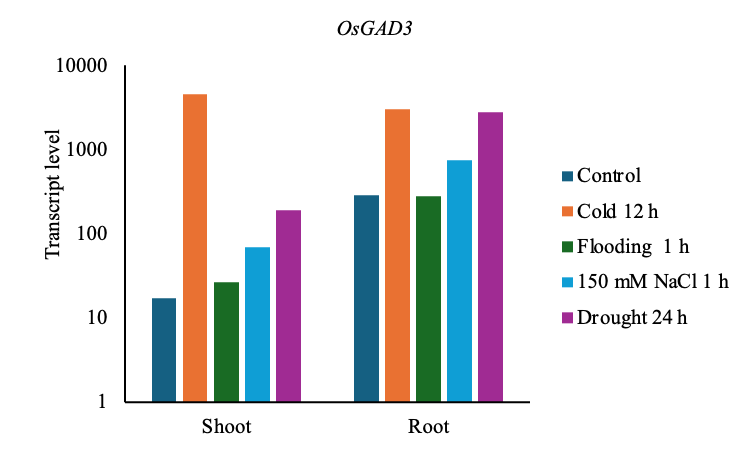


**Fig. S5 Analysis of *OsGAD1* and *OsGAD3* transcript levels in response to abiotic stress conditions.** The values indicate the abundance of *OsGAD1* and *OsGAD3* transcripts in shoot and root tissues, derived from mRNA-seq data retrieved from the TENOR database (https://tenor.dna.affrc.go.jp/).

**Fig. S6 Expression levels of stress-related genes derived from transcriptome analysis.** The bar chart illustrates the expression levels of various stress-related genes in WT Ni (wild-type), OsGAD1ΔC #5, OsGAD3ΔC #8, and Hybrid #78 control conditions. Expression levels are represented as log_2_ (FPKM+1) values.


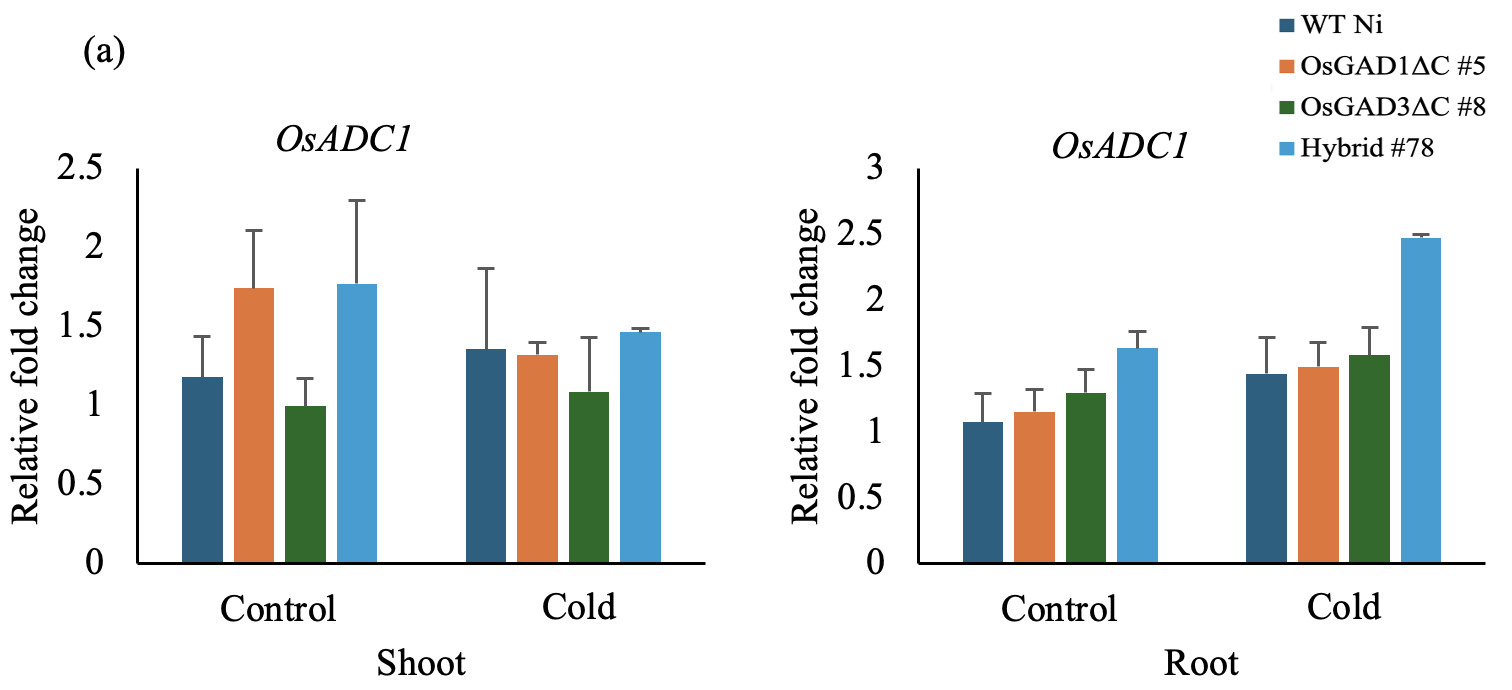

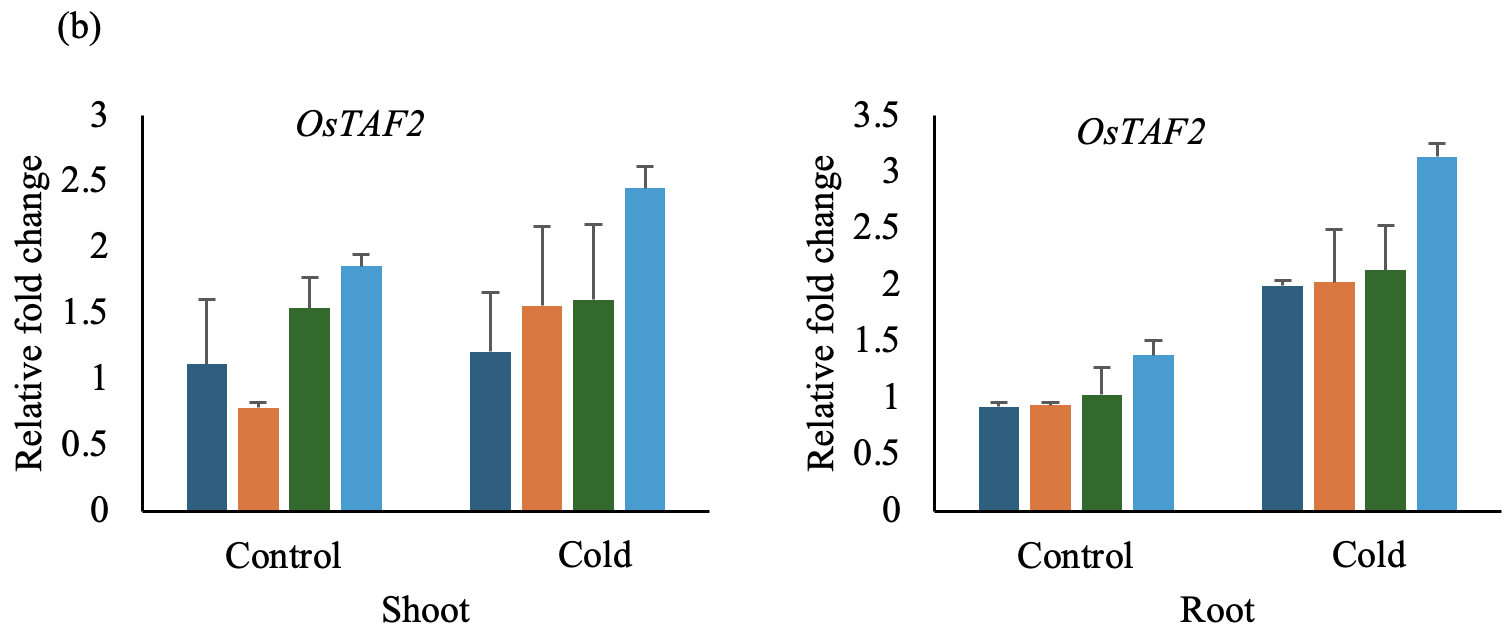

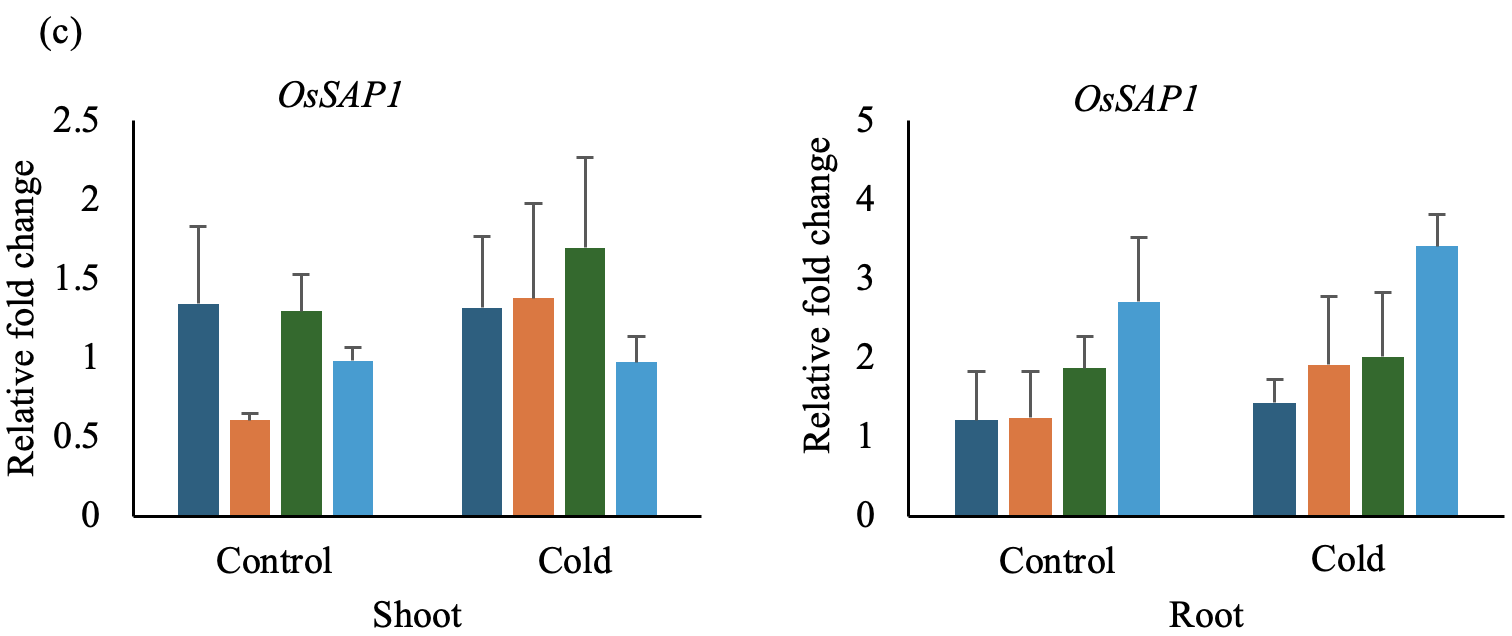


**Fig. S7 Relative expression of cold stress-related genes in rice seedlings**. (a) *OsADC*, (b) *OsTAF2*, and (c) *OsSAP1* expression in shoot and root tissues of WT Ni (wild-type), OsGAD1ΔC #5, OsGAD3ΔC #8, and Hybrid #78 in control conditions (without stress) followed by the exposure to cold (4°C) for 12 h. Bars represent the mean ± standard deviation (SD) (n=3) of relative fold change. Expression levels were analyzed using the 2^^-ΔΔCt^ method, where TATA-binding protein (TBP2) was used as an internal control.


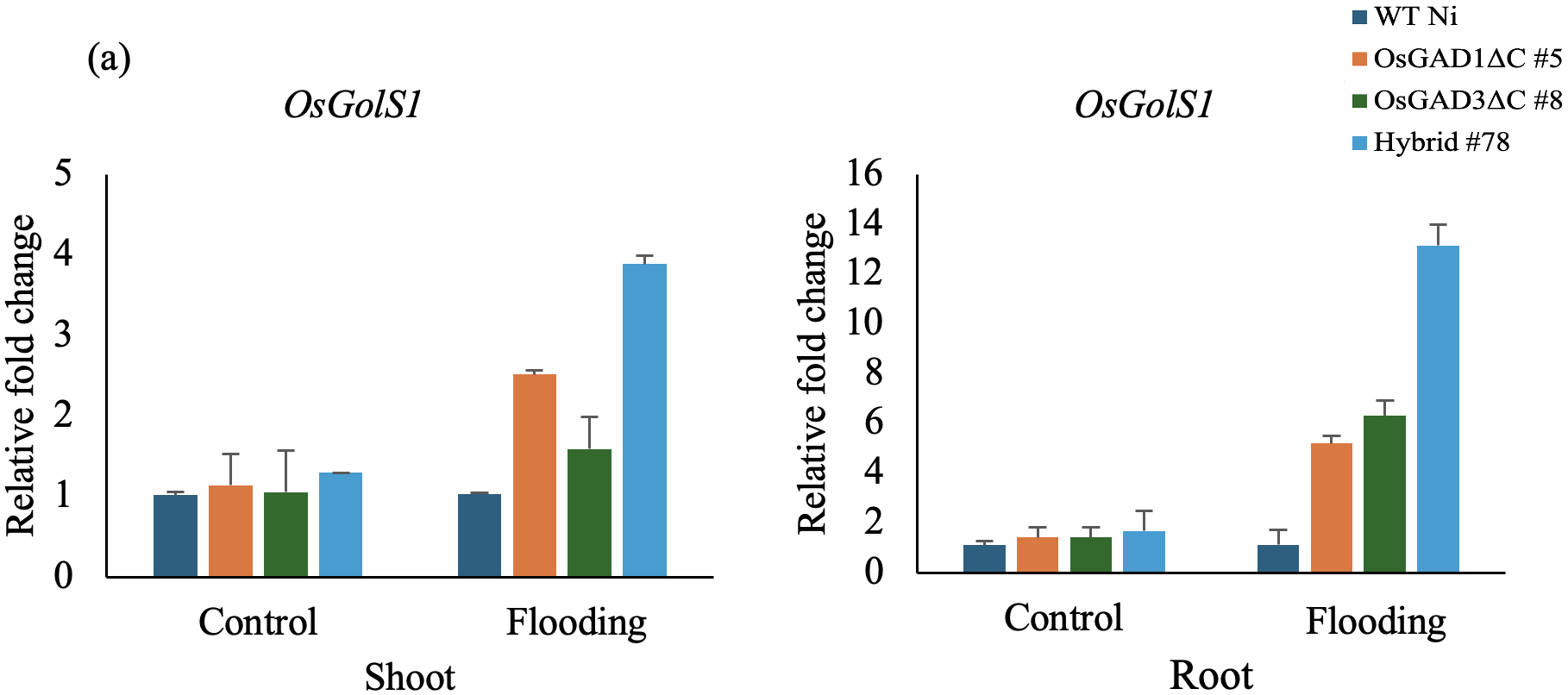

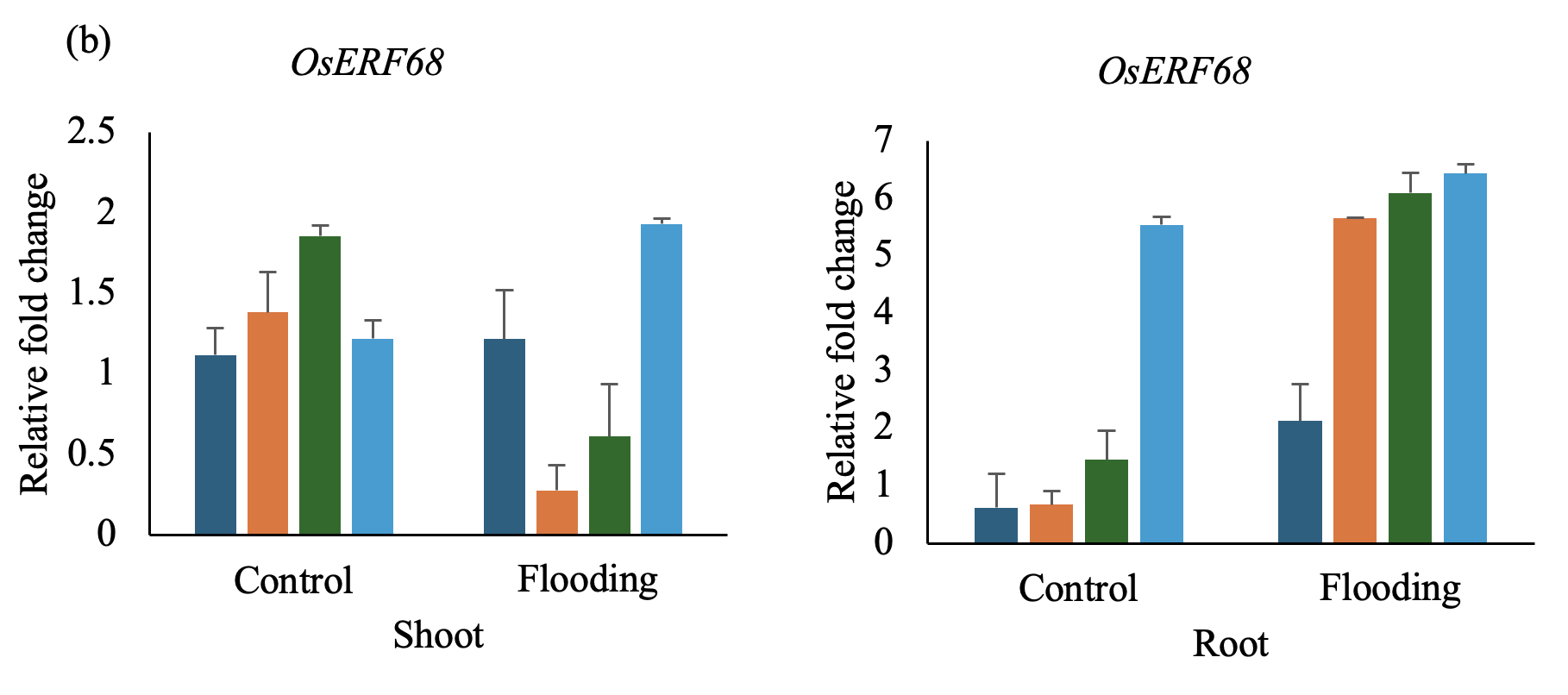

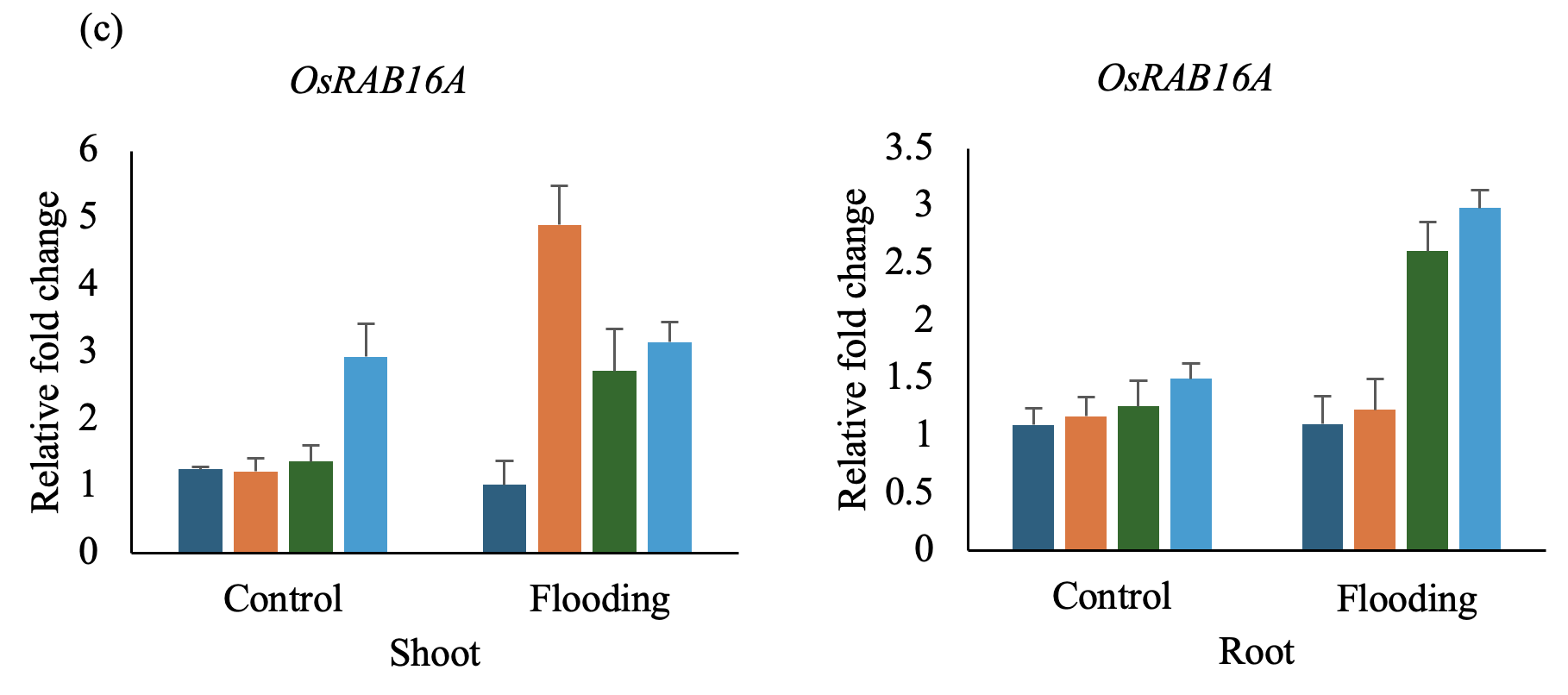


**Fig. S8** **Relative expression of flooding stress-related genes in rice seedlings**. (a) *OsGolS1*, (b) *OsERF68*, and (c) *OsRAB16A* expression in shoot and root tissues of WT Ni (wild-type), OsGAD1ΔC #5, OsGAD3ΔC #8, and Hybrid #78 in control conditions (without stress) followed by exposure to flooding conditions for 3 h. Bars represent the mean ± standard deviation (SD) (n=3) of relative fold change. Expression levels were analyzed using the 2^^-ΔΔCt^ method, where TATA-binding protein (TBP2) was used as an internal control.


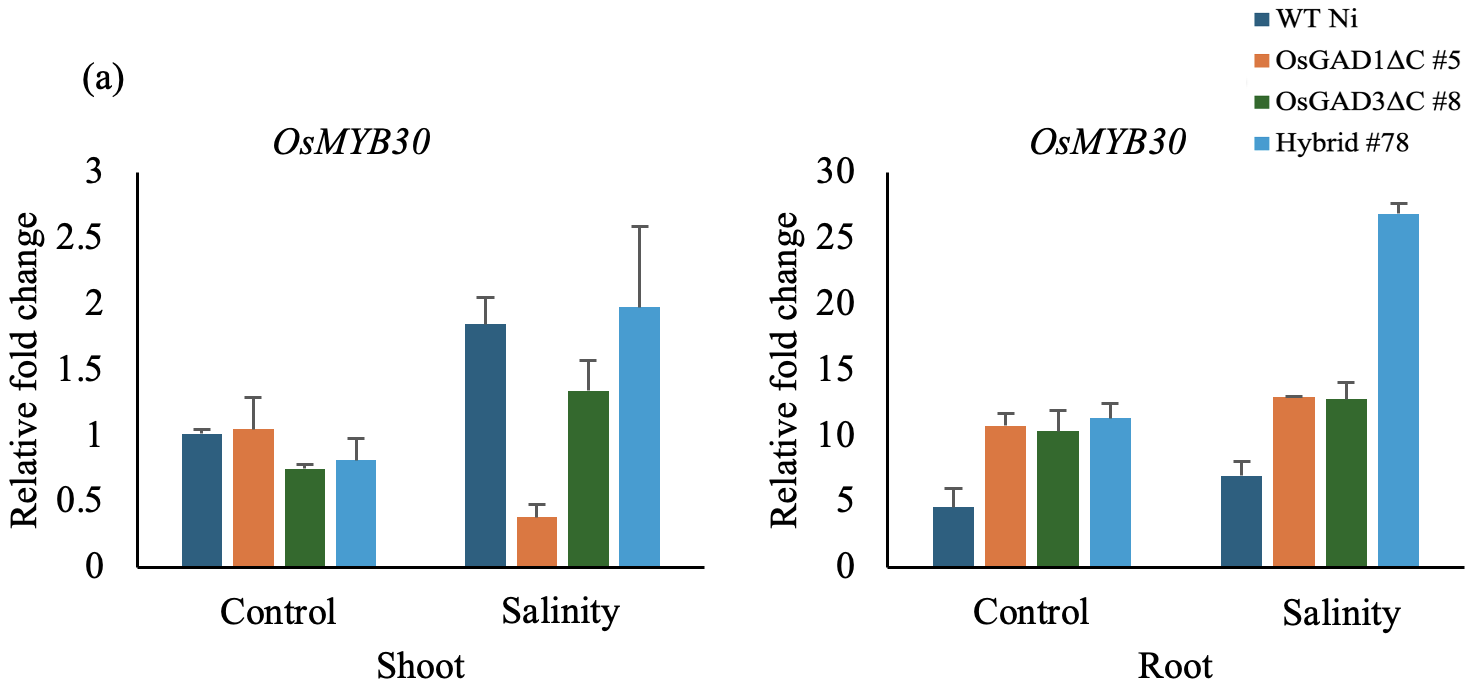

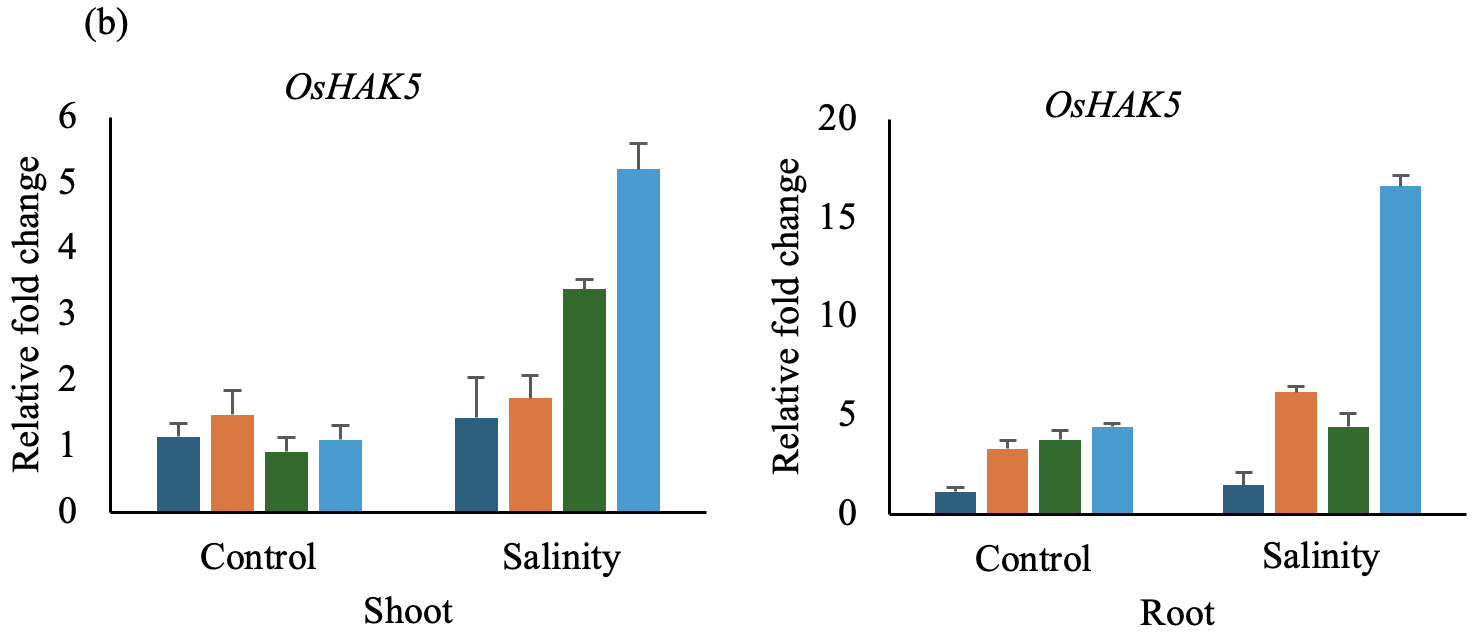

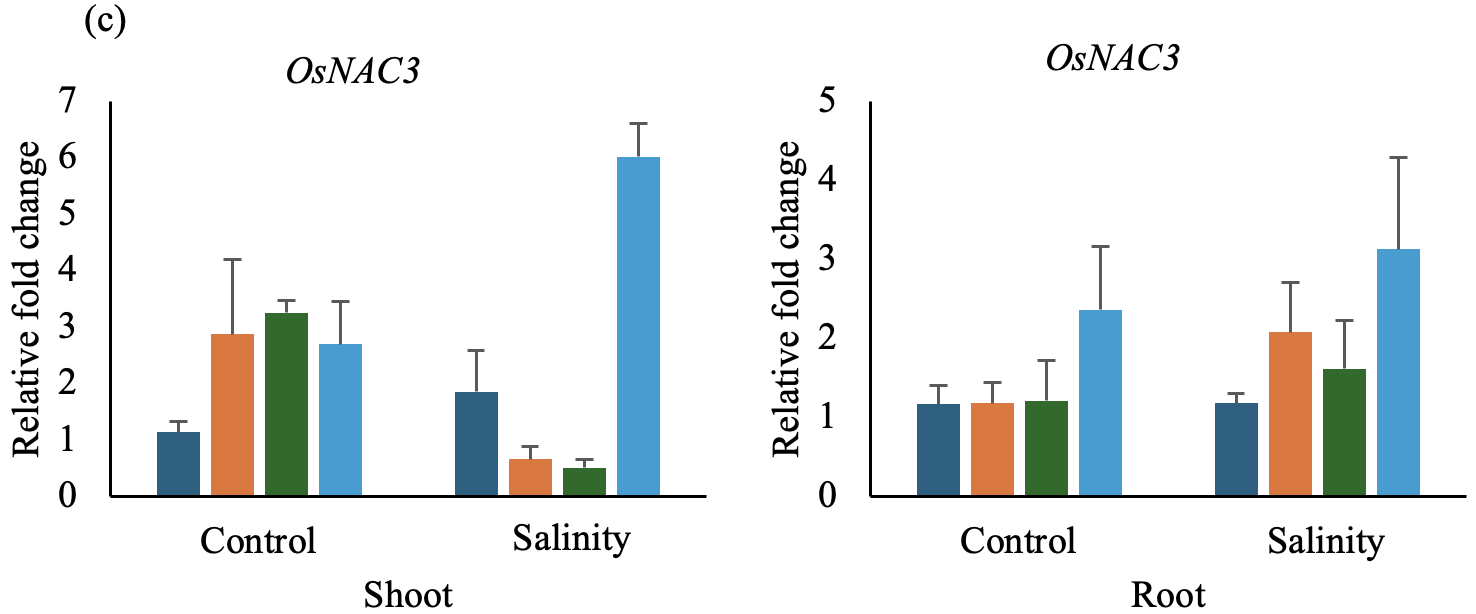


**Fig. S9** **Relative expression of salinity stress-related genes in rice seedlings**. (a) *OsMYB30*, (b) *OsHAK5*, and (c) *OsNAC3* expression in shoot and root tissue of WT Ni (wild-type), OsGAD1ΔC #5, OsGAD3ΔC #8, and Hybrid #78 in control conditions (without stress) followed by exposure to salinity condition (150 mM NaCl) for 3 h. Bars represent the mean ± standard deviation (SD) (n=3) of relative fold change. Expression levels were analyzed using the 2^^-ΔΔCt^ method, where TATA-binding protein (TBP2) was used as an internal control.


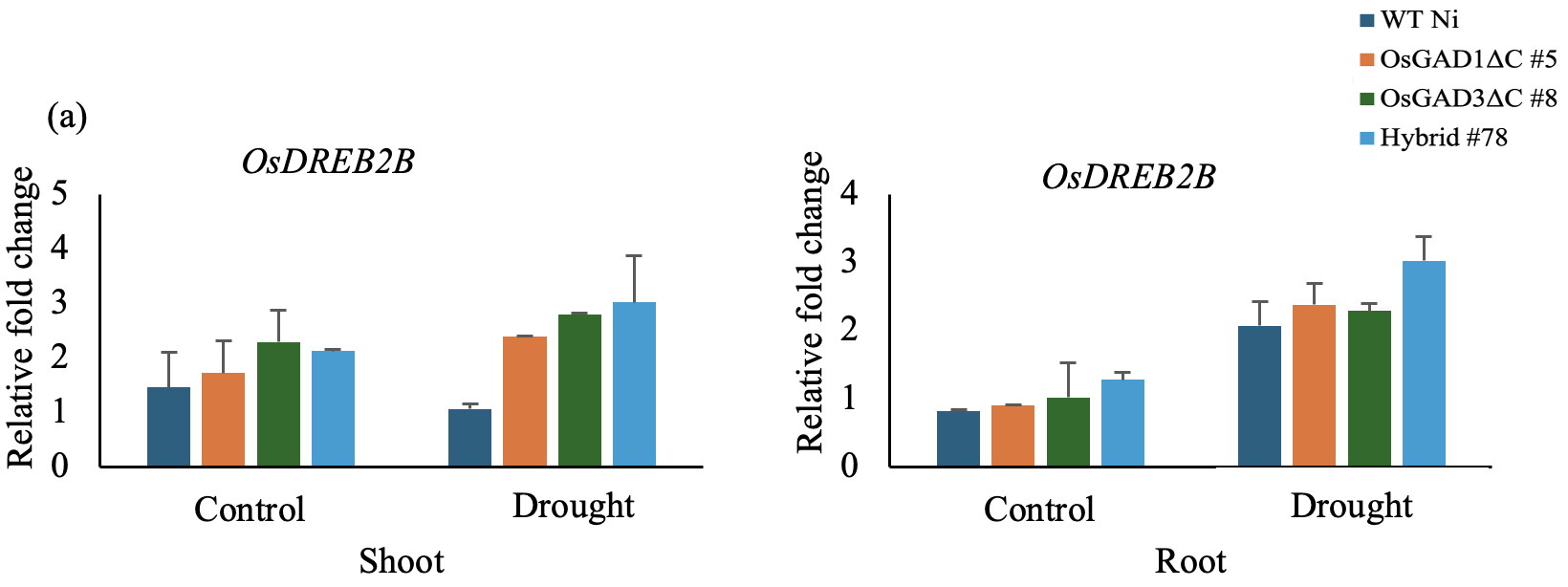

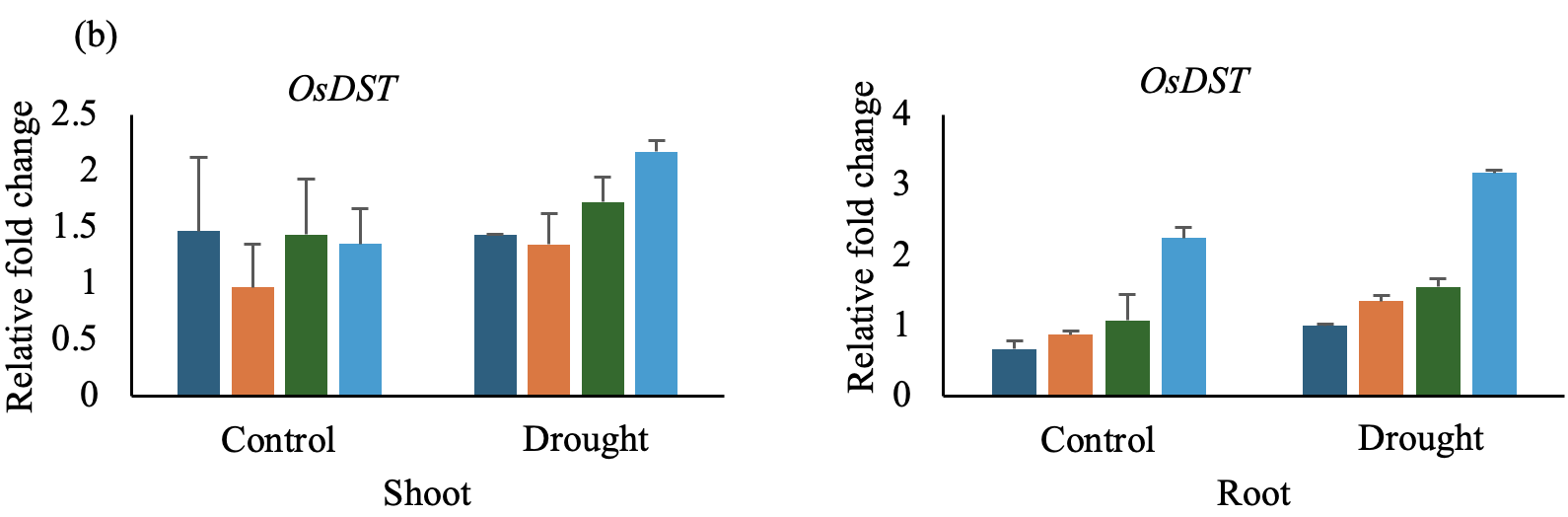

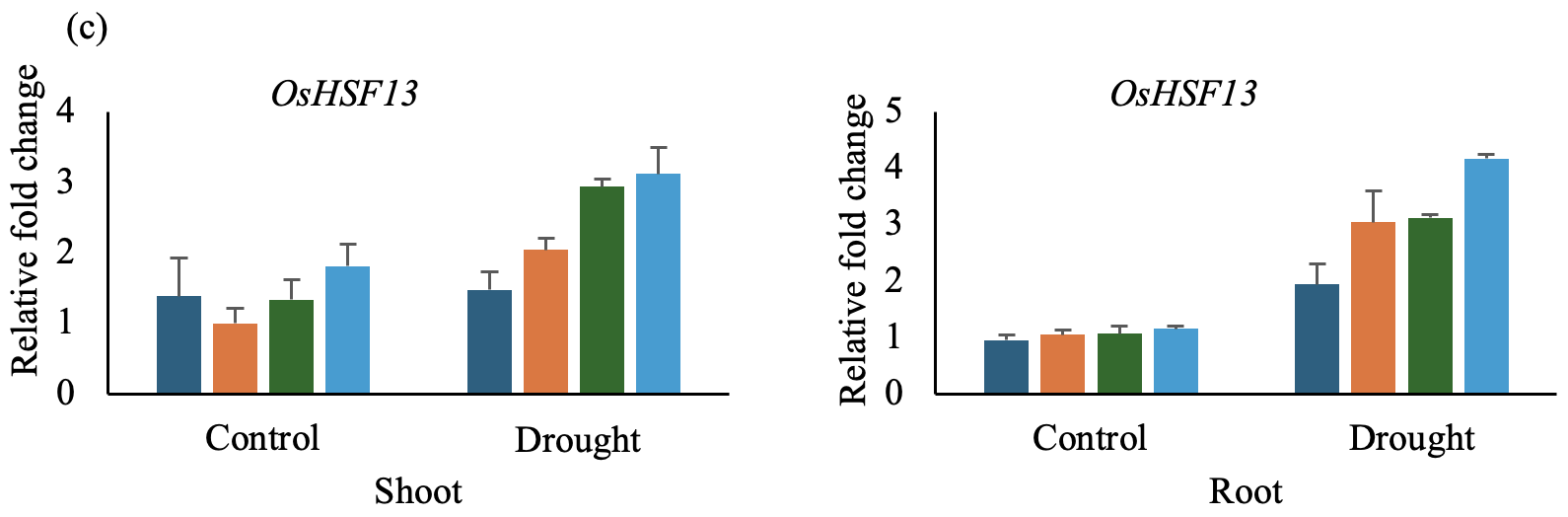

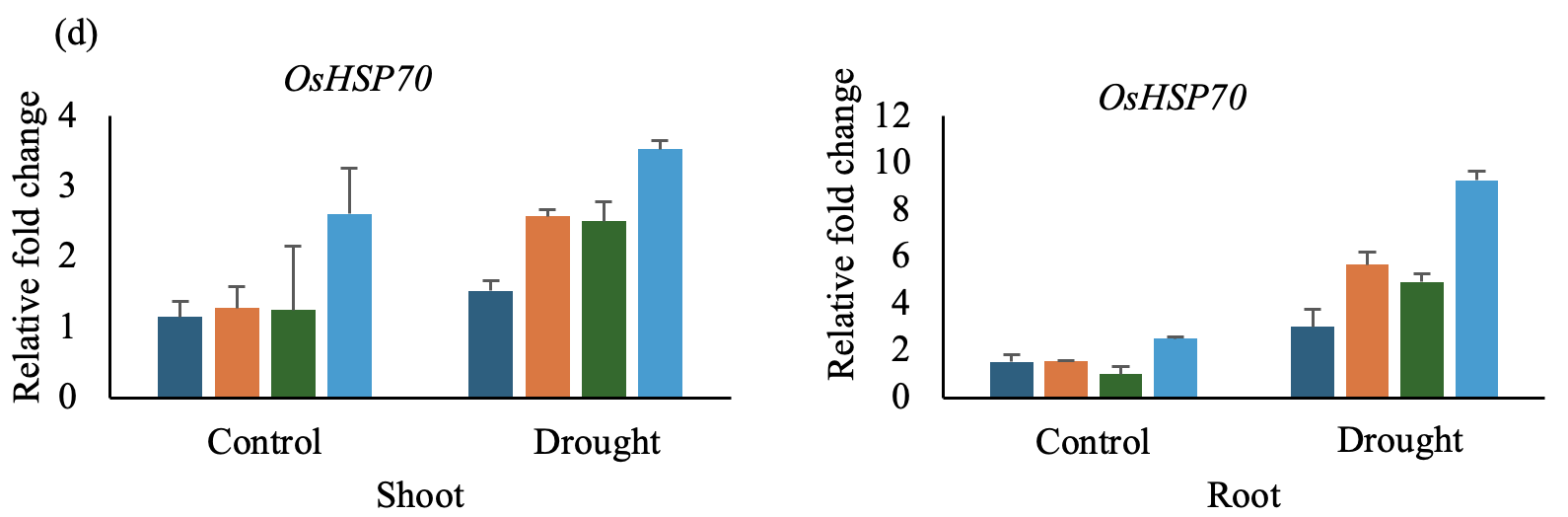


**Fig. S10** **Relative expression of drought stress-related genes in rice seedlings**. (a) *OsDREB2B*, (b) *OsDST*, (c) *OsHSF13*, and (d) *OsOsHSP70* expression in shoot and root tissues of WT Ni (wild-type), OsGAD1ΔC #5, OsGAD3ΔC #8, and Hybrid #78 in control conditions (without stress) followed by exposure to drought conditions for 24 h. Bars represent the mean ± standard deviation (SD) (n=3) of relative fold change. Expression levels were analyzed using the 2^^-ΔΔCt^ method, where TATA-binding protein (TBP2) was used as an internal control.
